# Supplementary material for: Transcriptional Activation by Oct4 Is Sufficient for the Maintenance and Induction of Pluripotency
Source: Cell Rep. 2012 Feb 23;1(2):99–109. doi: 10.1016/j.celrep.2011.12.002 (PMC3778438; doi:10.1016/j.celrep.2011.12.002)
Supplement: Table S2. Gene Ontology of Upregulated and Downregulated Genes in Oct4λVP2 Cell Line, Related to Figure 3 — Gene ontology (GO) annotations of upregulated and downregulated genes in Oct4λVP2 cell line in comparison to control cell lines with more than 2 fold change. Categories with enrichment < 2, FDR < 0.05 or contain less than 5 genes are not shown [file mmc2.pdf]

Table S2. Gene Ontology of Upregulated and Downregulated Genes in the Oct4λVP2 Cell Line, Related to Figure 3

Annotation	Enrichment ratio	p	FDR	Gene symbols
**Upregulated**[a]				
endopeptidase inhibitor activity	3.178	0	0	Serpinb1b,A2m,Timp4,Ambp,Serpinb6c,Cst9,Tfpi,Serpine1,Pzp,Pbp2,Cst13,Serpinb6b,Serpinb9b,Serpinb1a,Plxnc1,Papln,Serpinb1c,Wfikkn2,Vwf,Serpinb9,Lxn,Cst3,Pcsk1n,
protease inhibitor activity	3.151	0	0	Serpinb1b,A2m,Timp4,Ambp,Serpinb6c,Cst9,Tfpi,Serpine1,Pzp,Pbp2,Cst13,Serpinb6b,Serpinb9b,Serpinb1a,Plxnc1,Papln,Serpinb1c,Wfikkn2,Vwf,Serpinb9,Lxn,Cst3,Pcsk1n,
serine-type endopeptidase inhibitor activity	3.351	0	0	Serpinb1b,Ambp,Serpinb6c,Tfpi,Serpine1,Pzp,Pbp2,Serpinb6b,Serpinb9b,Serpinb1a,Plxnc1,Papln,Serpinb1c,Wfikkn2,Vwf,Serpinb9,Pcsk1n,
enzyme inhibitor activity	2.558	0	0	Serpinb1b,A2m,Timp4,Ambp,Serpinb6c,Ppp1r14d,Cst9,Tfpi,Serpine1,Pzp,Pbp2,Cst13,Serpinb6b,Serpinb9b,Serpinb1a,Plxnc1,Papln,Serpinb1c,Wfikkn2,Vwf,Serpinb9,Lxn,Cst3,Ppp1r1b,Pcsk1n,
alpha-type channel activity	2.067	0	0	Grid2,Aqp9,Pkd2l1,Grin1,Pkd1l2,Fxyd4,Gjb4,Aqp3,Gjb3,Cacna1g,Kcna5,Kcnc4,Scn3a,Clic3,Clca1,Grin2d,Scnn1b,Kcnn3,Ryr3,Itpr3,Kctd8,Aqp7,Gabrr1,Gjb5,Trpa1,Gjc1,Chrnb2,Clcnka,P2rx5,Kctd1,Gja7,Accn3,Trpm6,Kcns3,Kctd15,Kcnd1,Kcnj12,Cnga3,Kcnu1,
prostaglandin metabolism	9.238	0	0	Ptges,Ptgs1,Hpgd,Tnfrsf1a,
intracellular ligand-gated ion channel activity	9.238	0	0	Clca1,Ryr3,Itpr3,Cnga3,
prostanoid metabolism	9.238	0	0	Ptges,Ptgs1,Hpgd,Tnfrsf1a,
regulation of body fluids	3.943	0	0	Nfe2,Tfpi,Thbd,Proz,Mst1,Rab27a,Clcnka,Vwf,Il10rb,Entpd2,
angiogenesis	3.403	0	0.001	Serpine1,Tie1,Kdr,Angpt2,Robo4,Ang1,Mmp19,Edg1,Crhr2,Eng,Epas1,Vegfc,
blood vessel morphogenesis	3.233	0	0.001	Serpine1,Tie1,Kdr,Angpt2,Robo4,Ang1,Mmp19,Edg1,Crhr2,Eng,Epas1,Ptprj,Vegfc,
protein polymerization	4.715	0	0.001	Tubb3,Tubb2a,Gas7,Tuba3,Tubal3,Ttll3,Tubb2b,
connexon complex	6.218	0	0.001	Gjb4,Gjb3,Gjb5,Gjc1,Gja7,
connexon channel activity	6.218	0	0.001	Gjb4,Gjb3,Gjb5,Gjc1,Gja7,
gap-junction forming channel activity	6.218	0	0.001	Gjb4,Gjb3,Gjb5,Gjc1,Gja7,
structural constituent of eye lens	6.218	0	0.001	Lim2,Cryge,Crygf,Cryab,Crygb,
hemostasis	3.829	0	0.002	Nfe2,Tfpi,Thbd,Proz,Mst1,Rab27a,Vwf,Il10rb,Entpd2,
cell growth	3.355	0.0001	0.002	Emp1,Fhl1,Nov,Htra1,Tgfb1,Igfbp2,Igfbp1,Lefty2,Socs7,Creg1,Igfbp5,
icosanoid metabolism	4.85	0.0001	0.003	Alox12e,Ptges,Ptgs1,Ggtla1,Hpgd,Tnfrsf1a,
regulation of cell size	3.175	0.0002	0.004	Emp1,Fhl1,Nov,Htra1,Tgfb1,Igfbp2,Igfbp1,Lefty2,Socs7,Creg1,Igfbp5,
regulation of lymphocyte activation	3.804	0.0002	0.004	Il7,Efnb1,Fcgr2b,Prkcq,Tgfb1,Zap70,Hmgb3,Nfam1,
regulation of cell activation	3.804	0.0002	0.004	Il7,Efnb1,Fcgr2b,Prkcq,Tgfb1,Zap70,Hmgb3,Nfam1,

Annotation	Enrichment ratio	p	FDR	Gene symbols
response to wounding	2.209	0.0003	0.005	Tfpi,Ggtla1,Fcgr2b,Csf3r,Thbd,Rel,Il17b,Prkcq,Tlr3,Proz,Tgfb1,Tnfrsf1a,Mst1,Rab27a,Ccr4,Il1f8,Gadd45g,Vwf,Lcp1,Fn1,Il10rb,Entpd2,
regulation of cell migration	4.042	0.0003	0.005	Tie1,Robo4,Itgb3,Cxcr4,Sst,Pax6,Lama1,
blood vessel development	2.765	0.0003	0.006	Serpine1,Tie1,Kdr,Angpt2,Robo4,Ang1,Mmp19,Edg1,Crhr2,Eng,Epas1,Ptprj,Vegfc,
striated muscle thick filament	8.084	0.0004	0.006	Myom2,Mybpc3,Obscn,
A band	8.084	0.0004	0.006	Myom2,Mybpc3,Obscn,
photoreceptor cell development	8.084	0.0004	0.006	Nr2e3,Rpgrip1,Pax6,
inorganic anion exchanger activity	8.084	0.0004	0.006	Slc4a1,Slc4a2,Slc4a11,
regulation of B cell activation	5.052	0.0004	0.006	Il7,Fcgr2b,Tgfb1,Hmgb3,Nfam1,
insulin-like growth factor binding	5.052	0.0004	0.005	Nov,Htra1,Igfbp2,Igfbp1,Igfbp5,
gap junction	5.052	0.0004	0.005	Gjb4,Gjb3,Gjb5,Gjc1,Gja7,
lymphocyte proliferation	3.902	0.0004	0.006	Il7,Efnb1,Fcgr2b,Prkcq,Zap70,Cxcr4,Ccnd3,
regulation of cell motility	3.902	0.0004	0.006	Tie1,Robo4,Itgb3,Cxcr4,Sst,Pax6,Lama1,
vasculature development	2.694	0.0005	0.007	Serpine1,Tie1,Kdr,Angpt2,Robo4,Ang1,Mmp19,Edg1,Crhr2,Eng,Epas1,Ptprj,Vegfc,
UDP-galactosyltransferase activity	5.879	0.0005	0.007	Abo,A4galt,B3galnt1,Wdfy3,
regulation of lymphocyte differentiation	5.879	0.0005	0.006	Il7,Zap70,Hmgb3,Nfam1,
blood coagulation	3.495	0.0006	0.007	Tfpi,Thbd,Proz,Mst1,Rab27a,Vwf,Il10rb,Entpd2,
wound healing	3.163	0.0008	0.009	Tfpi,Thbd,Proz,Mst1,Rab27a,Vwf,Fn1,Il10rb,Entpd2,
regulation of locomotion	3.65	0.0009	0.01	Tie1,Robo4,Itgb3,Cxcr4,Sst,Pax6,Lama1,
coagulation	3.316	0.001	0.011	Tfpi,Thbd,Proz,Mst1,Rab27a,Vwf,Il10rb,Entpd2,
positive regulation of lymphocyte proliferation	5.389	0.001	0.011	Il7,Efnb1,Prkcq,Zap70,
response to drug	5.389	0.001	0.011	Phf11,D14Ertd668e,Abp1,Drd1a,
tubulin-tyrosine ligase activity	6.929	0.001	0.011	Ttll6,Ttll3,Ttl,
regulation of angiogenesis	6.929	0.001	0.011	Serpine1,Tie1,Crhr2,
water channel activity	6.929	0.001	0.01	Aqp9,Aqp3,Aqp7,
oxidoreductase activity, acting on the CH-OH group of donors, NAD or NADP as acceptor	2.502	0.0013	0.012	Akr1c18,Hsd17b2,Hpgd,Ldhal6b,Idh2,Rnasel,Akr1c13,Akr1b7,Me3,Tdh,Gpd1,H6pd,Adh1,
growth factor binding	3.429	0.0015	0.014	Il1rl2,Nov,Htra1,Igfbp2,Igfbp1,Il10rb,Igfbp5,
growth	2.382	0.0016	0.015	Emp1,Ptch1,Fhl1,Nov,Htra1,Tgfb1,Igfbp2,Igfbp1,Lefty2,Gdf3,Socs7,Lgmn,Creg1,Igfbp5,

Annotation	Enrichment ratio	p	FDR	Gene symbols
enzyme linked receptor protein signaling pathway	2.104	0.0017	0.015	Hhip,Kit,Rspo2,Clnk,Kdr,Ptprb,Smad6,Angpt2,Htra1,Ddr2,Erbb3,Tgfb1,Ptprcap,Wfikkn2,Ephb4,Gfra1,Ptpre,Eng,Ptprj,
ligand-gated ion channel activity	2.552	0.0017	0.014	Grid2,Grin1,Clca1,Grin2d,Scnn1b,Ryr3,Itpr3,Gabrr1,Chrnb2,P2rx5,Accn3,Cnga3,
lymphocyte activation	2.552	0.0017	0.014	Il7,Efnb1,Fcgr2b,Prkcq,Sfpi1,Tgfb1,Zap70,Cxcr4,Gadd45g,Hmgb3,Nfam1,Ccnd3,
transmembrane receptor protein tyrosine phosphatase signaling pathway	4.974	0.0018	0.014	Ptprb,Ptprcap,Ptpre,Ptprj,
chymotrypsin activity	2.287	0.0019	0.015	Tmprss7,Klk11,Tmprss11d,Prss35,Klk1b26,Htra1,Tpsg1,Proz,Klk1b1,Tmprss5,Mst1,Klk1b24,Klk10,Klk13,C1r,
muscle development	2.333	0.0021	0.017	Myl7,Tnnt2,Efhb,Myom2,Mybpc3,Fhl1,Cryab,Mylpf,Tgfb1,Hspb2,Chat,Myod1,Utrn,Csrp3,
fatty acid metabolism	2.266	0.0021	0.016	Alox12e,Adipoq,Ptges,Scd1,Ptgs1,Acoxl,Ggtla1,Fads2,Hpgd,Tnfrsf1a,Slc27a3,Fads1,Ucp3,Prkag1,Scap,
water transporter activity	6.063	0.0023	0.018	Aqp9,Aqp3,Aqp7,
carbon-nitrogen ligase activity, with glutamine as amido-N-donor	6.063	0.0023	0.017	Cps1,Gdpd2,Gpnmb,
anion exchanger activity	6.063	0.0023	0.017	Slc4a1,Slc4a2,Slc4a11,
water transport	6.063	0.0023	0.017	Aqp9,Aqp3,Aqp7,
anion:anion antiporter activity	6.063	0.0023	0.017	Slc4a1,Slc4a2,Slc4a11,
pregnancy	6.063	0.0023	0.017	Thbd,Mst1,Sult1e1,
bicarbonate transporter activity	6.063	0.0023	0.017	Slc4a1,Slc4a2,Slc4a11,
fluid transport	6.063	0.0023	0.017	Aqp9,Aqp3,Aqp7,
eye development (sensu Vertebrata)	4.042	0.0024	0.017	Lim2,Cryab,Crygb,Mitf,Pax6,
regulation of lymphocyte proliferation	4.042	0.0024	0.017	Il7,Efnb1,Fcgr2b,Prkcq,Zap70,
galactosyltransferase activity	4.042	0.0024	0.017	Abo,B3gnt3,A4galt,B3galnt1,Wdfy3,
nucleotide catabolism	4.619	0.0029	0.02	Entpd3,Nt5e,Upp1,Entpd2,
transmembrane receptor protein tyrosine kinase activity	2.939	0.0031	0.022	Kit,Rspo2,Kdr,Ddr2,Erbb3,Ncam1,Ror1,Ephb4,
cytokine binding	2.745	0.0034	0.022	Il1rl2,Il18r1,Il6ra,Tnfrsf1a,Tnfrsf8,Ccr4,Cxcr4,Cxcr3,Il10rb,
T cell proliferation	3.849	0.0034	0.023	Efnb1,Prkcq,Zap70,Cxcr4,Ccnd3,
interaction between organisms	3.849	0.0034	0.022	Edn2,Thbd,Mst1,Sult1e1,Ppp1r1b,
oxidoreductase activity, acting on CH-OH group of donors	2.284	0.004	0.025	Akr1c18,Hsd17b2,Hpgd,Ldhal6b,Idh2,Rnasel,Akr1c13,Akr1b7,Me3,Tdh,Gpd1,H6pd,Adh1,

Annotation	Enrichment ratio	p	FDR	Gene symbols
tissue kallikrein activity	5.389	0.0045	0.027	Klk1b26,Klk1b1,Klk1b24,
photoreceptor cell differentiation	5.389	0.0045	0.027	Nr2e3,Rpgrip1,Pax6,
physiological interaction between organisms	5.389	0.0045	0.027	Thbd,Mst1,Sult1e1,
phospholipid binding	2.978	0.0052	0.031	Hip1,Plekha2,Sytl4,Mfge8,Pclo,Cpne6,Anxa9,
heparin binding	2.978	0.0052	0.031	Abp1,Pcolce2,Ncam1,Lipc,Gpnmb,Fn1,Col5a1,
transmembrane receptor protein kinase activity	2.598	0.0056	0.033	Kit,Rspo2,Kdr,Ly6g6e,Ddr2,Erbb3,Ncam1,Ror1,Ephb4,
immune cell activation	2.282	0.0057	0.033	Il7,Efnb1,Fcgr2b,Prkcq,Sfpi1,Tgfb1,Zap70,Cxcr4,Gadd45g,Hmgb3,Nfam1,Ccnd3,
cell activation	2.256	0.0064	0.037	Il7,Efnb1,Fcgr2b,Prkcq,Sfpi1,Tgfb1,Zap70,Cxcr4,Gadd45g,Hmgb3,Nfam1,Ccnd3,
glutathione transferase activity	4.042	0.0067	0.038	Gsta3,Gstp1,Gsta2,Gsto1,
trypsin activity	2.055	0.0069	0.039	Tmprss7,Klk11,Tmprss11d,Prss35,Klk1b26,Htra1,Tpsg1,Proz,Klk1b1,Tmprss5,Mst1,Klk1b24,Klk10,Klk13,C1r,
chloride transporter activity	4.85	0.0079	0.043	Slc4a1,Slc4a2,Slc4a11,
symporter activity	2.377	0.0081	0.044	Slc6a7,Slc15a1,Slc5a1,Slc6a13,Slc28a1,Slc6a4,Slc23a1,Slc23a2,Slc12a7,Slc5a2,
cell surface	2.28	0.0082	0.044	Hhip,Ly6c,Ly6a,Kit,Thy1,Fcgr2b,Robo4,Jam4,Itgb3,Mfge8,Nfam1,
T cell activation	2.76	0.0094	0.05	Il7,Efnb1,Prkcq,Zap70,Cxcr4,Gadd45g,Ccnd3,
**Downregulated**[b]				
transcription factor activity	2.27	0	0	Rhox11,Egr1,Cart1,Hod,Trpv6,Asb9,Foxd1,Foxc1,Sox4,Egr2,Wt1,Stat4,Esrrg,Gata4,Meis1,Tcfec,Gata6,Grhl2,Trpc3,Pou5f1,Gata3,Sox17,Hmx3,Msx1,Foxg1,Jun,Ovol1,Isl2,Hlf,Mpdz,Ripk4,Rxrg,Thrb,Hlxb9,Emx2,Tbx15,Grm1,Hmx1,Trpc7,Atf5,Pou6f1,Foxa3,Vax2,Nkx6-3,Tbx21,Bach2,Foxp1,Rhox10,Foxf2,Tsc22d3,Nkx3-1,Dmrt2,Cebpb,Ar,Irx3,Onecut1,Dlx4,Hoxa7,Mkx,Asb4,Emx1,Runx1t1,Satb1,Hoxb4,Hand1,Dmrt3,Foxq1,Irx2,Spib,Gata5,Taf13,Nr4a2,Ipf1,Lef1,Foxl2,Foxc2,Rai14,Irf8,Dmrtb1,Irx5,Jund1,Nr2c1,Pou3f3,Hes6,Barx1,Trim30,En2,Invs,Gbx2,Nr2f1,Apol6,Elf1,Lhx2,Prox1,Tbx6,
cell differentiation	2.037	0	0	Mcf2,Egr1,Sema6a,Ephb1,Foxd1,Flt1,Xdh,Egr2,Wt1,Zic1,Artn,Fst,Efnb2,Gata3,Slitrk3,Jun,Fgf5,Sema3c,Sfrp4,Emx2,Ifnz,Reln,Angpt1,Chrdl2,Smo,Bcl11a,Crb3,Cebpb,Shh,Onecut1,Amot,Rhob,Ifrd1,Mdfi,Mcoln3,Emx1,Tnfrsf12a,Fgf15,Runx1t1,Kras,Sema3e,Nog,Bmp5,Nr4a2,Lef1,Irf8,Hes6,Cebpa,Ptk7,Spry1,Ascl2,En2,Dcamkl1,Bmpr1b,Gbx2,Rnf6,Dock2,Fgf8,Prox1,Kif5c,Tbx6,
system development	2.227	0	0	Mcf2,Aph1a,Sema6a,Ephb1,Foxd1,Egr2,Wt1,Zic1,Artn,Met,Efnb2,Hmx3,Msx1,Slitrk3,Lmo4,Fgf5,Sema3c,Emx2,Reln,Smo,Cebpb,Agtr1a,Shh,Emx1,Tnfrsf12a,Sema3e,Nog,Nr4a2,Cables1,Hes6,Ptk7,Spry1,Ascl2,En2,Dcamkl1,Gbx2,Nr2f1,Fgf8,Lhx2,Kif5c,
epithelial cell differentiation	8.435	0	0	Xdh,Wt1,Jun,Crb3,Ptk7,
sex differentiation	4.898	0	0	Wt1,Fst,Wnt4,Dmrt2,Ar,Shh,Dmrt3,Dmrtb1,Spo11,
behavior	2.789	0	0	Itga8,Egr2,Zic1,Artn,Met,Cmtm8,Reln,Scn8a,Ccl20,Amot,Gnao1,Il1b,Vdac1,Mcoln3,Cyr61,Tshr,Prkca,Prk

Annotation	Enrichment ratio	p	FDR	Gene symbols
				ce,Cxcl16,Astn1,Dock2,
organ morphogenesis	2.101	0	0	Hod,Flt1,Id1,Wt1,Gata4,Gata6,Wnt5a,Fst,Efnb2,Hmx3,Id2,Thbs1,Thrb,Wnt2,Wnt4,Angpt1,Tgfa,Smo,Cebpb,Ar,Shh,Amot,Rhob,Hsd11b1,Hoxa7,Tnfrsf12a,Fgf15,Hoxb4,Hand1,Cyr61,Wnt7a,Lef1,Spo11,Cebpa,Spry1,Htr2b,Fgf8,Igf2,
appendage morphogenesis	5.368	0	0	Sp8,Wnt5a,Lrp5,Wnt9a,Shh,Wnt7a,Fgf8,
limb morphogenesis	5.368	0	0	Sp8,Wnt5a,Lrp5,Wnt9a,Shh,Wnt7a,Fgf8,
appendage development	5.368	0	0	Sp8,Wnt5a,Lrp5,Wnt9a,Shh,Wnt7a,Fgf8,
nervous system development	2.116	0	0	Mcf2,Sema6a,Ephb1,Foxd1,Egr2,Zic1,Artn,Met,Efnb2,Hmx3,Msx1,Slitrk3,Lmo4,Fgf5,Sema3c,Emx2,Reln,Smo,Cebpb,Shh,Emx1,Tnfrsf12a,Sema3e,Nog,Nr4a2,Cables1,Hes6,Ptk7,Ascl2,En2,Dcamkl1,Gbx2,Nr2f1,Lhx2,Kif5c,
central nervous system development	3.426	0	0	Zic1,Met,Hmx3,Msx1,Emx2,Reln,Smo,Shh,Emx1,Nog,En2,Nr2f1,Lhx2,
cell migration	2.656	0	0	Ephb1,Foxd1,Flt1,Artn,Lrp5,Reln,Shh,Amot,Il1b,Tnfrsf12a,Fgf15,Lama4,Kiss1r,Prkca,Srgap1,Gbx2,Nr2f1,Astn1,Dock2,Kif5c,
vasculature development	3.067	0	0	Flt1,Id1,Efnb2,Thbs1,Angpt1,Tgfa,Smo,Shh,Amot,Rhob,Tnfrsf12a,Hand1,Lama4,Cyr61,
locomotory behavior	2.942	0	0	Artn,Cmtm8,Reln,Scn8a,Ccl20,Amot,Gnao1,Il1b,Mcoln3,Cyr61,Tshr,Prkca,Cxcl16,Astn1,Dock2,
brain development	3.501	0	0	Met,Hmx3,Msx1,Emx2,Reln,Shh,Emx1,Nog,En2,Nr2f1,Lhx2,
embryonic development	2.477	0	0	Sp8,Flt1,Gata4,Wnt5a,Hmx3,Lmo4,Lrp5,Cebpb,Ar,Wnt9a,Shh,Amot,Lama4,Cyr61,Wnt7a,Lef1,Cebpa,Ptk7,Ascl2,Fgf8,Tbx6,
BMP signaling pathway	6.025	0	0.001	Fst,Chrdl2,Bmper,Nog,Bmpr1b,
frizzled-2 signaling pathway	5.623	0.0001	0.001	Wnt5a,Wnt2,Wnt4,Wnt9a,Wnt7a,
blood vessel morphogenesis	3.067	0.0001	0.001	Flt1,Id1,Thbs1,Angpt1,Tgfa,Smo,Shh,Amot,Rhob,Tnfrsf12a,Hand1,Cyr61,
Wnt receptor signaling pathway	2.924	0.0001	0.001	Wnt5a,Sfrp4,Lrp5,Wnt2,Wnt4,Wnt9a,Kremen2,Fzd6,Fzd1,Dkk3,Wnt7a,Lef1,Csnk1e,
blood vessel development	2.885	0.0002	0.002	Flt1,Id1,Thbs1,Angpt1,Tgfa,Smo,Shh,Amot,Rhob,Tnfrsf12a,Hand1,Lama4,Cyr61,
embryonic limb morphogenesis	4.601	0.0002	0.002	Sp8,Wnt5a,Lrp5,Wnt9a,Shh,Wnt7a,
embryonic appendage morphogenesis	4.601	0.0002	0.002	Sp8,Wnt5a,Lrp5,Wnt9a,Shh,Wnt7a,
dorsal/ventral pattern formation	5.272	0.0002	0.003	Sp8,Smo,Shh,Bmpr1b,Lhx2,
locomotion	2.256	0.0002	0.003	Ephb1,Foxd1,Flt1,Artn,Lrp5,Reln,Shh,Amot,Il1b,Tnfrsf12a,Fgf15,Lama4,Kiss1r,Tshr,Prkca,Srgap1,Gbx2,Nr2f1,Astn1,Dock2,Kif5c,
fat cell differentiation	8.435	0.0002	0.003	Cebpb,Runx1t1,Cebpa,
tissue development	2.257	0.0005	0.005	Foxc1,Wt1,Gata3,Ovol1,Ank,Angpt1,Smo,Shh,Onecut1,Mcoln3,Epb4.1l5,Foxq1,Ptger4,Spry1,Bmpr1b,Fgf8,Lhx2,Prox1,Tbx6,
cell motility	2.176	0.0007	0.006	Ephb1,Foxd1,Flt1,Artn,Lrp5,Reln,Shh,Amot,Il1b,Tnfrsf12a,Fgf15,Lama4,Kiss1r,Prkca,Srgap1,Gbx2,Nr2f1

Annotation	Enrichment ratio	p	FDR	Gene symbols
				,Astn1,Dock2,Kif5c,
localization of cell	2.176	0.0007	0.006	Ephb1,Foxd1,Flt1,Artn,Lrp5,Reln,Shh,Amot,Il1b,Tnfrsf12a,Fgf15,Lama4,Kiss1r,Prkca,Srgap1,Gbx2,Nr2f1,Astn1,Dock2,Kif5c,
morphogenesis of a branching structure	4.686	0.0007	0.006	Flt1,Agtr1a,Shh,Egf,Cyr61,
enzyme linked receptor protein signaling pathway	2.195	0.0008	0.007	Ephb1,Flt1,Epha1,Fst,Angpt1,Chrdl2,Dok2,Bmper,Grb10,Nog,Prkca,Bmp5,Ptprk,Stxbp4,Ptprs,Plat,Bmpr1b,Ptprt,Ptpru,
gonad development	4.439	0.0011	0.01	Wt1,Fst,Wnt4,Ar,Spo11,
development of primary sexual characteristics	4.439	0.0011	0.01	Wt1,Fst,Wnt4,Ar,Spo11,
angiogenesis	2.859	0.0012	0.011	Flt1,Id1,Thbs1,Angpt1,Tgfa,Amot,Rhob,Tnfrsf12a,Hand1,Cyr61,
cell-cell signaling	2.017	0.0013	0.011	Pdyn,Glrb,Chrnb1,Egr2,Wnt5a,Gabra4,Wnt2,Gabra2,Wnt4,Wnt9a,Tph2,Shh,Prima1,Vdac1,Gabra3,Gja3,Wnt7a,Nr4a2,Spry1,Glra2,Fgf8,Pnoc,
urogenital system development	3.749	0.0016	0.012	Aph1a,Wt1,Agtr1a,Shh,Nog,Spry1,
metanephros development	4.217	0.0017	0.013	Aph1a,Wt1,Agtr1a,Nog,Spry1,
potassium channel activity	2.239	0.0024	0.018	Kctd12b,Kcna1,Kcnmb2,Kctd4,Kcnk1,Kctd14,Kcne3,Kcnj3,Kcnmb4,Kcnh8,Kcnk6,Kcnt2,Kcna7,Kcnh5,Kctd16,
GABA receptor activity	3.49	0.0029	0.021	Glrb,Gabra4,Gabra2,Gabra3,Gpr156,Glra2,
growth factor activity	2.181	0.0034	0.023	Areg,Artn,Nrg4,Fgf5,Tgfa,Il3,Il1b,Fgf15,Egf,Fgf21,Fgf13,Bmp5,Inha,Fgf8,Igf2,
protein homodimerization activity	3.374	0.0038	0.025	Cebpb,Cebpd,Runx1t1,Hand1,Dgkh,Cebpa,
pattern specification	2.144	0.0041	0.026	Sp8,Flt1,Fst,Emx2,Lrp5,Smo,Shh,Hoxa7,Hoxb4,Cyr61,Nog,Bmp5,Lef1,Bmpr1b,Lhx2,
axon guidance	3.028	0.0044	0.027	Ephb1,Foxd1,Artn,Shh,Tnfrsf12a,Gbx2,Kif5c,
embryonic morphogenesis	2.617	0.0051	0.029	Sp8,Wnt5a,Hmx3,Lmo4,Lrp5,Wnt9a,Shh,Wnt7a,Ptk7,
potassium ion transport	2.029	0.006	0.034	Kctd12b,Kcna1,Kcnmb2,Slc9a7,Kctd4,Kcnk1,Kctd14,Kcne3,Kcnj3,Kcnmb4,Kcnh8,Kcnk6,Kcnt2,Kcna7,Kcnh5,Kctd16,
rhythmic process	3.163	0.0062	0.034	Egr2,Hlf,Prokr1,Csnk1e,Spo11,Per2,
negative regulation of signal transduction	3.514	0.0063	0.034	Chrdl2,Bmper,Dkk3,Prkca,Elf1,
kidney development	3.514	0.0063	0.034	Aph1a,Wt1,Agtr1a,Nog,Spry1,
steroid hormone receptor activity	2.88	0.0066	0.035	Esrrg,Rxrg,Thrb,Ar,Nr4a2,Nr2c1,Nr2f1,
ligand-dependent nuclear receptor activity	2.88	0.0066	0.035	Esrrg,Rxrg,Thrb,Ar,Nr4a2,Nr2c1,Nr2f1,
mesoderm development	3.969	0.0072	0.038	Ovol1,Epb4.1l5,Lhx2,Tbx6,

Annotation	Enrichment ratio	p	FDR	Gene symbols
sulfotransferase activity	3.067	0.0077	0.038	Chst11,Chst1,Hs3st3b1,Chst7,Hs3st1,Sult1b1,
neurotransmitter receptor activity	2.2	0.0081	0.039	Glrb,Chrnb1,Oprk1,Prokr1,Htr1b,Gabra4,Gabra2,Gabra3,Sort1,Kiss1r,Tacr2,Glra2,
neurotransmitter binding	2.2	0.0081	0.039	Glrb,Chrnb1,Oprk1,Prokr1,Htr1b,Gabra4,Gabra2,Gabra3,Sort1,Kiss1r,Tacr2,Glra2,
regulation of signal transduction	2.279	0.0116	0.054	Reln,Chrdl2,Bmper,Dkk3,Prkca,Spred2,Cnksr2,Spry1,Elf1,Dock2,

[a] Gene ontology (GO) annotations of upregulated genes in Oct4λVP2 cell line with more than 2 fold change. Categories with enrichment <2, FDR<0.05 or contain less than 5 genes are not shown.

[b] Gene ontology (GO) annotations of downregulated genes in Oct4λVP2 cell line with more than 2 fold change. Categories with enrichment <2, FDR<0.05 or contain less than 5 genes are not shown.
