# Supplementary material for: Transcriptional Activation by Oct4 Is Sufficient for the Maintenance and Induction of Pluripotency
Source: Cell Rep. 2012 Feb 23;1(2):99–109. doi: 10.1016/j.celrep.2011.12.002 (PMC3778438; doi:10.1016/j.celrep.2011.12.002)
Supplement: Table S4. List of Antibodies, Related to Figures 5, 7, and S4 — The table lists the source and working dilution for each antibody used for Western blotting (WB), Immunofluorescence (IF), and Fluorescence-activated cell sorting (FACS). [file mmc4.pdf]

Table S4. List of Antibodies Used, Related to Figures 4, S4, S5, and S8

	**Protocol / Dilution**			
Protein	**WB**	**IF**	**FACS**	**Supplier/Reference**
Oct4		1 in 100		Santa Cruz (sc-9081)
Nanog		1 in 100		Chambers *et al* 2003
Sox2		1 in 1000		Santa Cruz (sc-17320)
E-cadherin			1 in 1000	Santa Cruz Biotechnolgy (sc-52328)
SSEA1			1 in 1000	DSHB
Anti-Rabbit- Alexa Fluor (2nd)		1 in 1000		Invitrogen (Alexa fluor-488)
Anti-Mouse- Alexa Fluor (2nd)		1 in 1000		Invitrogen (Alexa fluor-546)
Oct4	1:1000			Santa Cruz (sc-5279)
Nanog	1:1000			R&D (AF2729)
β-Tubulin	1:1000			Sigma-Aldrich (T2200)
Anti-Rabbit HRP	1:1000			Cell signalling (7074)
Anti-Goat HRP	1:2000			R&D (HAF109)
Anti-Mouse HRP	1:1000			Cell signalling (7076)
