# Supplementary material for: Transcriptional Activation by Oct4 Is Sufficient for the Maintenance and Induction of Pluripotency
Source: Cell Rep. 2012 Feb 23;1(2):99–109. doi: 10.1016/j.celrep.2011.12.002 (PMC3778438; doi:10.1016/j.celrep.2011.12.002)
Supplement: Table S5. List of qRT-PCR Primers, Related to Figures 3, 4, 5, 6, 7, 8, and S2 — The table lists the sequence and the qRT-PCR condition for each primer sets used. qRT-PCR was performed using LightCycler FastStart DNA MasterPLUS SYBR Green1 (SYBR) or universal probe library (UPL) mix from Roche. [file mmc5.pdf]

Table S5. Primer Sequences and qRT-PCR Conditions, Related to Figures 3, S2, S4, S5, S6, S7, and S8

Gene	**species**	**Forward primer**	**Reverse primer**	**RT-qPCR assay**	**Annealing temperature**
Gsc	X.*laevis*	GATGCCGCCAGTGCCTC	TGCAGCTCAGTTCGTGACAAA	**SYBR**	**55^{0}C**
Bmp4	X.*laevis*	AGCCCACTAAGGATGTGGTG	GCTGCTGAGGTTGAACACAA	**SYBR**	**55^{0}C**
Odc	X.*laevis*	GCCATTGTGAAGACCTCTCTCCATTC	TTCGGGTGATTCCTTGCCAC	**SYBR**	**55^{0}C**
Pou5f1	M. *musculus*	GGCGTTCTCTTTGGAAAGGTGTTC	CTCGAACCACATCCTTCTCT	**SYBR**	**58^{0}C**
Nanog	M. *musculus*	ATGAAGTGCAAGCGGTGGCAGAAA	CCTGGTGGAGTCACAGAGTAGTTC	**SYBR**	**63^{0}C**
Klf4	M. *musculus*	CGGGAAGGGAGAAGACACT	GAGTTCCTCACGCCAACG	**SYBR**	**58^{0}C**
Sox2	M. *musculus*	GGCGGCAACCAGAAGAACAG	GCTTGGCCTCGTCGATGAAC	**SYBR**	**58^{0}C**
Rex1	M. *musculus*	ACGAGTGGCAGTTTCTTCTTGGGA	TATGACTCACTTCCAGGGGGCACT	**SYBR**	**58^{0}C**
Fgf4	M. *musculus*	CCGGTTCTTCGTGGCTATGA	CTTACTGAGGGCCATGAACATACC	**SYBR**	**58^{0}C**
E-Cad	M. *musculus*	AGACTTTGGTGTGGGTCAGG	CATGCTCAGCGTCTTCTCTG	**SYBR**	**58^{0}C**
Nodal	M. *musculus*	GGCGTACATGTTGAGCCTCT	GCCTGGTGGAAAATGTCAAT	**SYBR**	**60^{0}C**
Bmp4	M. *musculus*	CAACCCAATTATGGGCTGGC	CCACAATCCAATCATTCCAGC	**SYBR**	**58^{0}C**
TBra	M. *musculus*	GTGACTGCCTACCAGAATGA	ATTGTCCGCATAGGTTGGAG	**SYBR**	**60^{0}C**
Wnt3	M. *musculus*	CGCTCAGCTATGAACAAGCA	GGTGTTTCTCCACCACCATC	**SYBR**	**60^{0}C**
Tbx4	M. *musculus*	ACACCTTCCCAACTCAGAGG	CTTGCAGGGCAAGTCCAG	**SYBR**	**58^{0}C**
Sprouty2	M. *musculus*	GAGAGGGGTTGGTGCAAAG	CTCCATCAGGTCTTGGCAGT	**UPL (3)**	**58^{0}C**
Gata4	M. *musculus*	GCCTGCGGCCTCTACATGAA	CAGGACCTGCTGGCGTCTTA	**SYBR**	**60^{0}C**
Gata6	M. *musculus*	GGTCTCTACAGCAAGATGAATGG	TGGCACAGGACAGTCCAAG	**UPL(40)**	**58^{0}C**
Mixl1	M. *musculus*	AGTTGCTGGAGCTCGTCTTC	AGGGCAATGGAGGAAAACTC	**SYBR**	**60^{0}C**
Foxa2	M. *musculus*	CATCCGACTGGAGCAGCTA	GCGCCCACATAGGATGAC	**SYBR**	**58^{0}C**
Cdx2	M. *musculus*	GGAAGCCAAGTGAAAACCAG	CTTGGCTCTGCGGTTCTG	**SYBR**	**58^{0}C**
Tbp	M. *musculus*	GGGGAGCTGTGATGTGAAGT	CCAGGAAATAATTCTGGCTCA	**SYBR**	**58^{0}C**
Sdf2	M. *musculus*	TTCTCACGACGTGCGCTAT	TCATCCACAGAGGTCACACC	**SYBR**	**58^{0}C**
Msx2	M. *musculus*	AGGAGCCCGGCAGATACT	GTTTCCTCAGGGTGCAGGT	**UPL (70)**	**58^{0}C**
Fgfr1	M. *musculus*	CGAATTGGAGGCTACAAGGT	GAAGGCACCACAGAATCCAT	**UPL(1)**	**58^{0}C**
Otx2	M. *musculus*	GACTGCAGGGCAGAGACG	GGTAGATTTGGAGTGACGGAAC	**SYBR**	**58^{0}C**
Dkk1	M. *musculus*	CCGGGAACTACTGCAAAAAT	GGTTTTCAATGATGCTTTCCTC	**UPL (76)**	**58^{0}C**
Endo Oct4	M. *musculus*	CCAACGAGAAGAGTATGAGGC	GTGCTTTTAATCCCTCCTCAG	**SYBR**	**58^{0}C**
Endo Sox2	M. *musculus*	TCTGTGGTCAAGTCCGAGGC	TTCTCCAGTTCGCAGTCCAG	**SYBR**	**58^{0}C**
Endo Klf4	M. *musculus*	GGCGAGAAACCTTACCACTGT	TACTGAACTCTCTCTCCTGGCA	**SYBR**	**58^{0}C**
Endo cMyc	M. *musculus*	TCAAGCAGACGAGCACAAGC	TACAGTCCCAAAGCCCCAGC	**SYBR**	**58^{0}C**
Total	M. *musculus*	CCTAGTGCTGCATGAGGAGA	TCTTCCTCATCTTCTTGCTCTTC	**SYBR**	**58^{0}C**

cMyc					
Eras	M. *musculus*	GCCCCTCATCAGACTGCTAC	GCAGCTCAAGGAAGAGGTGT	**SYBR**	**58^{0}C**
Essrb	M. *musculus*	TGGCAGGCAAGGATGACAGA	TTTACATGAGGGCCGTGGGA	**SYBR**	**58^{0}C**
Fbxo15	M. *musculus*	TGCCAATTGTTGGGAGTACA	CAGATGAGCCTCTAACAAACTTACTTC	**SYBR**	**58^{0}C**
Dppa4	M. *musculus*	AAGGGCTTTCCCAGAACAAT	TCCAGAGGAACTGTCACCTCA	**SYBR**	**58^{0}C**
Dppa5	M. *musculus*	ATTCGGGCTAAATGGATGC	TAGCTCCAGGGTCTTCATGG	**SYBR**	**58^{0}C**
Ecat1	M. *musculus*	GGCGAGCTGAGATTTGGATA	CCAGCCTCCAGAGCCTCTAT	**SYBR**	**58^{0}C**
Gbx2	M. *musculus*	GCTGCTCGCTTTCTCTGC	GCTGTAATCCACATCGCTCTC	**SYBR**	**58^{0}C**
Klf2	M. *musculus*	CTAAAGGCGCATCTGCGTA	TAGTGGCGGGTAAGCTCGT	**SYBR**	**58^{0}C**
Fgf5	M. *musculus*	AAAACCTGGTGCACCCTAGA	CATCACATTCCCGAATTAAGC	**SYBR**	**58^{0}C**
NANOG	Human	GAGAAGAGTGTCGCAAAAAAGGAAG	AGTTCTGGTCTTCTGTTTCTTGACC	**SYBR**	**59^{0}C**
DPPA4	Human	AAGCTGAGCTCCAAAGGCCAGAAAT	TTTGATTTGGGTAGGCAAAGGCACA	**SYBR**	**59^{0}C**
RPL7	Human	GTGAGCCCAAAGGTTCGAAAGGTGT	TCCATTGAAGATTTGACGAAGGCGA	**SYBR**	**59^{0}C**

"Endo" indicates primers designed to pick up expression of only the endogenous transcript, whereas "total" indicates primers that can detect both endogenous and the exogenously supplied reprogramming factor.
