# Supplementary material for: Transcriptional Activation by Oct4 Is Sufficient for the Maintenance and Induction of Pluripotency
Source: Cell Rep. 2012 Feb 23;1(2):99–109. doi: 10.1016/j.celrep.2011.12.002 (PMC3778438; doi:10.1016/j.celrep.2011.12.002)
Supplement: Document S1. Article plus Supplemental Information [file mmc6.pdf]

Cell Reports
Report

Cell PRESS
Open ACCESS

Transcriptional Activation by Oct4 Is Sufficient for the Maintenance and Induction of Pluripotency

Fella Hammachi,[1] Gillian M. Morrison,[1,6] Alexei A. Sharov,[2] Alessandra Livigni,[1] Santosh Narayan,[3] Eirini P. Papapetrou,[4,7] James O'Malley,[1] Keisuke Kaji,[1] Minoru S.H. Ko,[2] Mark Ptashne,[3,*] and Joshua M. Brickman[1,5,*]
[1]MRC Centre for Regenerative Medicine - Institute for Stem Cell Research, School of Biological Sciences, 5 Little France Drive, University of Edinburgh, EH16 4UU Edinburgh, UK
[2]Laboratory of Genetics, National Institute on Aging, NIH Biomedical Research Centre, 251 Bayview Boulevard, Suite 100, Baltimore, MD 21224, USA
[3]Molecular Biology Program, Memorial Sloan Kettering Cancer Center, 430E 67th St, New York, NY 10065, USA
[4]Center for Cell Engineering, Molecular Pharmacology and Chemistry Program, Memorial Sloan Kettering Cancer Center, 1275 York Avenue, New York, NY 10065, USA
[5]The Danish Stem Cell Centre - DanStem, University of Copenhagen, 3B Blegdamsvej, DK-2200 Copenhagen N
[6]Present address: The Wellcome Trust Centre for Stem Cell Research, University of Cambridge, Tennis Court Road, CB2 1QR Cambridge, UK
[7]Present address: Division of Hematology, University of Washington, 1705 NE Pacific St., Box 357710, Seattle, WA 98195, USA
*Correspondence: joshua.brickman@sund.ku.dk (J.M.B.), m-ptashne@mskcc.org (M.P.)
DOI 10.1016/j.celrep.2011.12.002

SUMMARY

Oct4 is an essential regulator of pluripotency in vivo and in vitro in embryonic stem cells, as well as a key mediator of the reprogramming of somatic cells into induced pluripotent stem cells. It is not known whether activation and/or repression of specific genes by Oct4 is relevant to these functions. Here, we show that fusion proteins containing the coding sequence of Oct4 or Xlpou91 (the *Xenopus* homolog of Oct4) fused to activating regions, but not those fused to repressing regions, behave as Oct4, suppressing differentiation and promoting maintenance of undifferentiated phenotypes in vivo and in vitro. An Oct4 activation domain fusion supported embryonic stem cell self-renewal in vitro at lower concentrations than that required for Oct4 while alleviating the ordinary requirement for the cytokine LIF. At still lower levels of the fusion, LIF dependence was restored. We conclude that the necessary and sufficient function of Oct4 in promoting pluripotency is to activate specific target genes.

INTRODUCTION

Cells that can differentiate into all adult cell types exist transiently in early embryos. When cultured in vitro, these pluripotent embryonic stem cells (ESCs) can self-renew indefinitely (Evans and Kaufman, 1981; Martin, 1981). Pluripotent, self-renewing cell lines, called induced pluripotent stem cells (iPSCs), can also be derived from somatic cells by transient ectopic expression of transcription factors that are normally expressed in ESCs (Takahashi and Yamanaka, 2006). The pluripotent state can be maintained in vitro by defined cytokines such as LIF (Smith et al., 1988; Williams et al., 1988) and BMP4 or serum (Ying et al., 2003) for mouse pluripotent cells. In particular, removal of LIF leads to spontaneous differentiation of mouse ESCs toward mesoderm and endoderm (Ying et al., 2003).

The class V Pou (PouV) transcription factor Oct4 is central to both the generation and the maintenance of iPSCs and ESCs. Thus, removal of Oct 4 from ESCs causes these cells to differentiate into trophectoderm and primitive endoderm (Hay et al., 2004; Niwa et al., 1998; Niwa et al., 2000), and of the original four factors that together enable iPSC formation, (Oct4, Klf4, Sox2, and c-Myc) (Takahashi and Yamanaka, 2006), only Oct4 is essential regardless of the source of somatic cell (Kim et al., 2009). In mouse embryos, Oct4 is initially expressed in the inner cell mass (ICM) of the blastocyst but persists in the pluripotent epiblast and becomes progressively restricted to the posterior region of the embryo, where progenitor cells remain throughout gastrulation (Palmieri et al., 1994). Oct4$^{-/-}$ embryos die at preimplantation stages as a result of the differentiation of pluripotent ICM progenitors to trophectoderm (Nichols et al., 1998). Oct4 homologs in nonmammalian species are also expressed in gastrulation-stage progenitors, where they act to block precocious differentiation (Cao et al., 2006; Lavial et al., 2007; Lunde et al., 2004; Morrison and Brickman, 2006; Reim et al., 2004). The *Xenopus laevis* PouV protein, Xlpou91, can support murine ESC self-renewal of Oct4 null cells, and *Oct4* mRNA can rescue some *Xenopus* PouV knockdown phenotypes (Morrison and Brickman, 2006). What gene-regulatory mechanisms are required to establish and maintain ESCs and iPSCs, and in particular, what role is played by Oct4?

Despite an abundance of data on Oct4 targets and phenotypes, it is still unclear how Oct4 acts as a transcription factor to regulate differentiation. Experiments with reporter genes in cell lines suggest that Oct4 can function as both an activator and a repressor of gene transcription (reviewed in Pan et al., 2002), and Oct4 has been found to be associated with both activator and repressor complexes (Ang et al., 2011; Bilodeau et al., 2009; Boyer et al., 2006; Pardo et al., 2010; Pasini et al., 2010; Yeap et al., 2009; Yuan et al., 2009).

Table 1. PouV Fusion Proteins

Fusion Protein	PouV Protein	Hinge	Transcriptional Regulatory Region	Repeats
Oct4λVP2	mOct4	λ C1 (91–132)	VP16 (413–460)	2
XlPou91λVP2	XlPou91	λ C1 (91–132)	VP16 (413–460)	2
OCT4-VP16	hOCT4	-	VP16 (425–455)	1
OCT4-VP16	hOCT4	-	VP16 (425–490)	1
OCT4-VP16 (3F)	hOCT4	-	VP16 (PADALDDFDLDML)	3
Oct4λEnR	mOct4	λ C1 (91–132)	EnR (168–281)	1
XlPou91λEnR	XlPou91	λ C1 (91–132)	EnR (168–281)	1
OCT4-HP1α	hOCT4	-	HP1α	1
OCT4-HP1β	hOCT4	-	HP1β	1
OCT4-HP1γ	hOCT4	-	HP1γ	1

The human and mouse Oct4 genes and the *Xenopus* homolog XlPou91 were variously fused to one or more activating regions from VP16, to a repressing region from the *Drosophila* Engrailed protein, and to one of each of the three intact isoforms of the human protein HP1. In some cases a sequence encoding a hinge region in lambda repressor c1 was inserted between Oct4 or XlPou91, added regulatory sequences, and repeated regulatory modules (Ohashi et al., 1994). PADALDDFDLDML is a single amino acid variant of an activating sequence found in VP16 (Baron et al., 1997). "Repeats" indicate the number of copies of the activation region in the corresponding fusion. The three OCT4-VP16 activation fusions are listed in the order of their presumed activation strengths, referred to in the text as "weak," "medium," and "strong." Abbreviations are as follows: m, murine; h, human; Xl, *Xenopus laevis*.

Genome-wide chromatin immunoprecipitation (ChIP) experiments, with both human and mouse ESCs, have detected Oct4 bound to numerous genes, some of which are expressed in ESCs and some of which are not (Boyer et al., 2005; Chen et al., 2008; Kim et al., 2008; Loh et al., 2006). Some of these genes become upregulated, and some downregulated, as ESCs differentiate in response to Oct4 knockdown (Sharov et al., 2008) (Loh et al., 2006). This finding has led to the suggestion that, in ESCs, Oct4/PouV and its partners Sox2 and Nanog simultaneously activate genes encoding components of a "pluripotency network" and, simultaneously, repress differentiation-specific genes (Bilodeau et al., 2009; Boyer et al., 2005; Boyer et al., 2006; Chen et al., 2008; Kim et al., 2008; Loh et al., 2006; Pasini et al., 2010). A contrasting suggestion is that Oct4 drives the expression of genes required for differentiation (and not for pluripotency) but this pathway is somehow blocked in ESCs by the conflicting effects of other genes (e.g., Nanog, Klf4, etc.) that attempt to promote differentiation along different pathways (Loh and Lim, 2011). One line of evidence purported to support this idea is that overexpression of Oct4, like its removal, causes ESCs to differentiate (Niwa et al., 2000).

Here, we study Oct4 derivatives designed to act exclusively as activators or repressors of transcription. Each protein bears the PouV DNA-recognition sequence fused to either a strong transcriptional activation region (VP16 and derivatives thereof) or a strong repression region (Engrailed or HP1). We find that the activator, but not the repressor, fusions (along with other transcription factors) drive iPSC formation. The activator fusions, but not the repressor fusions, maintain the growth and pluripotency of murine ESCs lacking wild-type Oct4, and the activator fusions uniquely block differentiation of multipotent progenitor cells in *Xenopus* embryos. While maintaining ESCs in place of Oct4, an Oct4-activator fusion can render the pluripotent state both LIF-independent and immune to an "overexpression" phenotype (apparently by strongly activating key genes). We suggest that the activity of Oct4 as an activator is sufficient for the induction of iPSC formation and the maintenance of ESCs and embryonic progenitors.

RESULTS

Converting PouV Proteins to Activators and Repressors

Table 1 shows a series of fusion proteins, each with the DNA-binding specificity of Oct4 or Xlpou91, designed to work exclusively as activators or as repressors. Genes encoding these proteins were constructed by fusing intact murine and human Oct4 and Xlpou91 sequences, separately, to an activating region derived from mammalian VP16; to the repressing region of the *Drosophila* protein Engrailed (EnR); and to the mammalian heterochromatin protein HP1. In some cases the flexible hinge region of lambda C1 was interposed between the PouV protein and attached regulatory regions, an approach that has been particularly effective at converting full-length homeodomain repressors to activators (Brickman et al., 2001; Brickman et al., 2000; Zamparini et al., 2006). As a control, we also made fusions in which the DNA-binding domain was damaged by the mutation V267P (Niwa et al., 2002). We tested the activities of these fusion proteins with reporters introduced into the previously described murine ESC line ZHBTc4 (Niwa et al., 2000). Both endogenous copies of Oct4 have been deleted from these cells, and an Oct4 transgene, under control of a tetracycline (Tc)-repressible promoter, has been added. The cells grow as ESCs in the absence of Tc, and addition of Tc causes them to differentiate into trophoblast and primitive endoderm cells. In the absence of Tc, an introduced reporter gene bearing Oct4 binding sites is expressed, but by 24 hr after the addition of Tc, the introduced reporter fails to be expressed. mRNA and protein measurements confirm that the added Oct4 gene was essentially silent at this time. Within 96 hr after the addition of Tc, the cells have differentiated (Morrison and Brickman, 2006; Niwa et al., 2002; Niwa et al., 2000).

We cotransfected ZHBTc4 ESCs 24 hr after the addition of Tc with, separately, one of two different Oct4 responsive reporter genes (Niwa et al., 2002) and one or another of the Pou proteins listed in Table 1. One reporter contains the Oct4- and Sox2-dependent enhancer of the *Fgf4* gene driving the TK promoter, and the other reporter contains six copies of a reiterated octamer-binding site. Both Oct4λVP2 and Xlpou91λVP2 efficiently activated both reporters, whereas Oct4λEnR and Xlpou91λEnR repressed basal expression. The fusion proteins neither activated nor repressed transcription from a reporter lacking any PouV protein-responsive elements. λVP2 and λEnR had little activity when fused to Oct4V267P (Niwa et al., 2002) (Figure S1).

Activator Fusion Proteins Block Differentiation In Vivo

We injected mRNA encoding Xlpou91, Xlpou91λEnR, or Xlpou91λVP2 into early *Xenopus* embryos and assessed, by in situ hybridization and RT-PCR, the expression of early lineage markers at gastrulation. We found that markers of early mesoderm and endoderm differentiation (Sox17, Mixer, and Goosecoid [Gsc]) were suppressed by Xlpou91 and Xlpou91λVP2 and were enhanced by Xlpou91λEnR. In contrast, the ventral marker BMP4, whose activity has been linked to ESC self-renewal (Morrison and Brickman, 2006; Ying et al., 2003), responded oppositely to these added factors (Figures 1A and S2A). The capacity of Xlpou91λEnR to induce the expression of differentiation markers implies that their induction is an indirect effect of inhibiting the PouV-supported network of undifferentiated gene expression.

Activator Fusion Proteins Rescue Oct4 Null ESCs

Figure 1B shows that exogenously added Xlpou91λVP2 and Oct4λVP2 maintained ZHBTc4 ESC growth in the absence of endogenous Oct4 (i.e., in the presence of Tc), whereas neither of the repressor fusions did so. The colonies depicted in the figure were generated by stable transfection of the ZHBTc4 ESCs with vectors expressing both the *Pac* gene (which renders cells resistant to puromycin) and one or another wild-type PouV proteins or PouV fusion protein under the control of the strong constitutive CAG promoter. The efficiency with which Oct4λVP2 supported Oct4 null ESCs (the rescue index, or normalized rescue efficiency) (Niwa et al., 2002) was equivalent to that obtained with wild-type Oct4. Although the rescue index for Xlpou91λVP2 was not as high as that obtained by Oct4λVP2, both proteins supported identical undifferentiated colony morphologies. Inspection of the rescued colonies suggests that Oct4λVP2 and Xlpou91λVP2 rendered the cells less likely to spontaneously differentiate than those rescued by wild-type Oct4 or Xlpou91. Thus, the latter displayed typical ESC colony morphology, with undifferentiated cells in the center and differentiated cells around the periphery (Figure 1C). In contrast, colonies rescued by the activator fusion proteins appeared undifferentiated throughout (Figure 1D). The mutant Oct4V267P, fused to either λVP2 or λEnR, was unable to support growth in the absence of Oct4 (Figure S2B). We also noted that colonies derived from stable transfection of ZHBTc4 with Oct4 or Xlpou91, in the absence of Tc, contained more differentiated cells than those in the presence of Tc (Figure 1C). This phenotype is consistent with the observation that overexpression (as well as underexpression) of Oct4 induces differentiation (Niwa et al., 2000). However, colonies derived from transfection of the activator forms of both proteins remained completely undifferentiated in the absence of Tc, a matter we return to below. Transfection of Oct4λEnR and Xlpou91λEnR into Oct4-expressing cells produced colonies composed largely of differentiated cells, as expected (Figure 1E). Similar completely undifferentiated colony morphologies were observed when Oct4λVP2 was introduced into the unrelated E14Tg2A ESC line (Figure S2C).

We further substantiated the rescue experiment just described by generating ZHBTc4 cell lines maintained by wild-type Oct4 and, separately, by Oct4λVP2 and by Xlpou91λVP2. Colonies like those in Figures 1C and 1D were expanded for at least five passages in the presence of constant puromycin and Tc. The Oct4λVP2- and Xlpou91λVP2-supported cultures had fewer differentiated cells than did those supported by wild-type Oct4 (Figure S4A). In both cases we examined multiple independent clonal cell lines to assure that ESC phenotypes were not the result of isolated karyotypic changes. As Oct4λVP2 and Xlpou91λVP2- expressing cell lines appeared identical, we focused on Oct4λVP2-supported cell lines. Four different Oct4λVP2-supported cell lines were also confirmed to have normal karyotypes (data not shown). We found that these cell lines self-renewed as efficiently as Oct4-supported lines, as assayed by assessment of the capacity of single cells to generate ESC colonies (Figures S4B and S4C). No difference in proliferation or plating efficiency was detected when these lines were compared to either ZHBTc4 or the unrelated ESC line E14Tg2A (Figure S4D). ESCs supported by Oct4λVP2, like ZHBTc4 and E14Tg2A cells, expressed the ESC transcription factors Nanog, Sox2, and Oct4 and the cell-surface markers *SSEA-1* and *E-cadherin* (Figure 3B, S4E, and S4F).

Activator Fusion Proteins Induce Pluripotency

As noted in the Introduction, Oct4 is typically required, along with other transcription factors, to induce reprogramming of somatic cells to iPSCs in vitro. We tested the ability of various PouV-activator fusions and -repressor fusions to substitute for wild-type Oct4 in such a reprogramming experiment. We were able to generate both human and murine stable iPSCs by using activator, but not, where tested, repressor fusions (Figures 2A and 2B). The mouse iPSCs in Figure 2A were generated with the use of a single *piggy*Bac transposon that bears the *Sox2*, *Klf4*, and *c-Myc* genes, as well as either wild-type Oct 4 or Oct4λVP2, all under control of a Tc-inducible promoter (Kaji et al., 2009). Although the timing of the appearance of ESC-like iPSC colonies after transfection and addition of the Tc analog Doxycycline (Dox) was comparable in the two cases, the Oct4λVP2 colonies grew faster than did their counterparts containing wild-type Oct4 (Figure 2A). The Oct4λVP2-derived iPSC colonies were expanded in the presence of Dox. As expected for iPSCs, growth became independent of the exogenous genes, as indicated by growth in the absence of Dox (Figures S5A and S5B). When Oct4λVP2-induced iPSCs were transplanted into adult mice, they formed teratomas containing derivatives of all three germ layers (Figure S5C), a further indication of pluripotency. To generate human iPSCs, we infected human fibroblasts with lentiviral vectors expressing the three additional factors (all human)

Cell PRESS
Open ACCESS

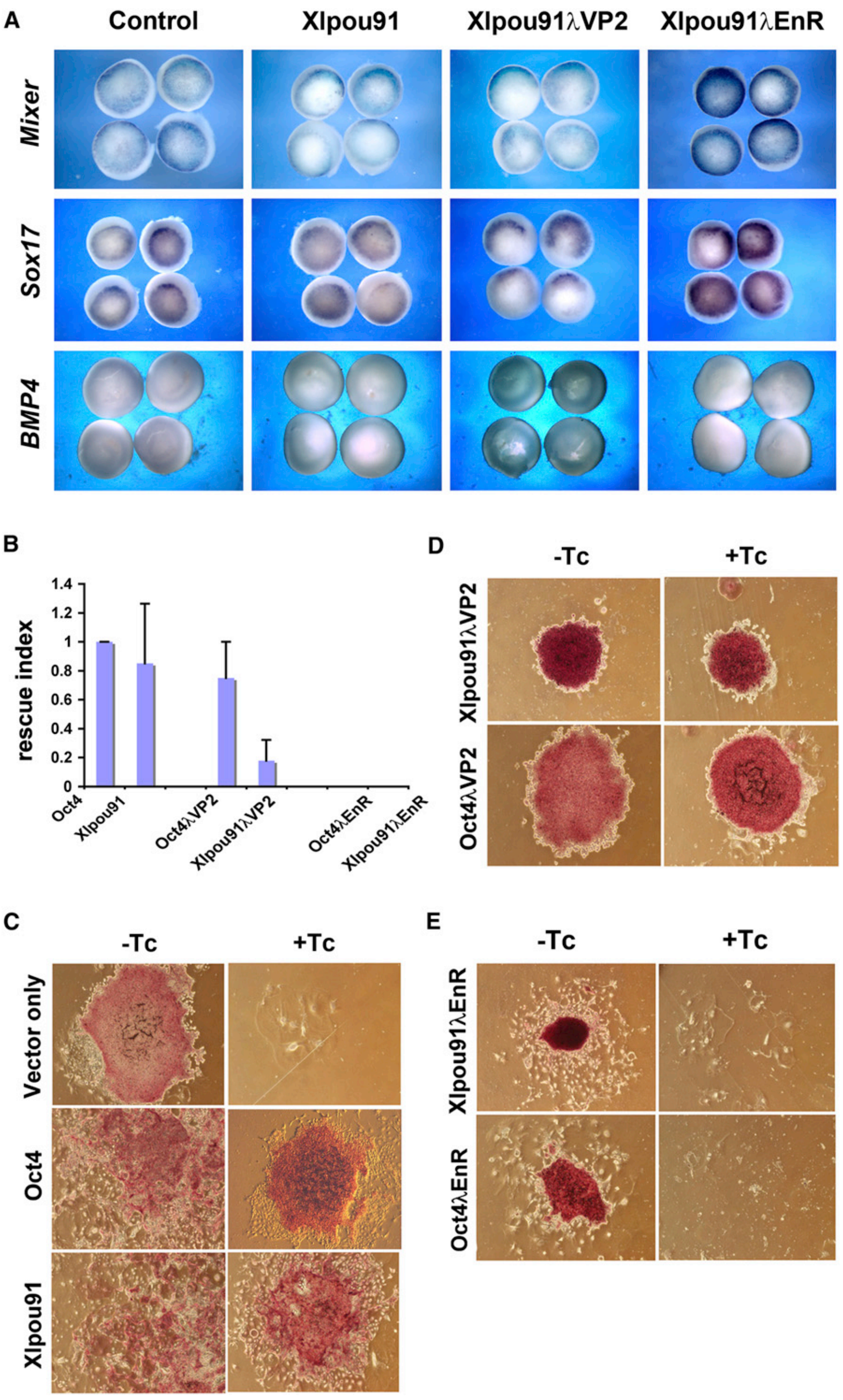

Figure 1. Actions of PouV-Activator and -Repressor Proteins

(A) In situ assays for the mRNA products of Mixer, Sox17, and BMP4 were performed on stage 10.25 embryos after injection of Xlpou91, Xlpou91λVP2, or Xlpou91λEnR mRNA at the two-cell stage into both blastomeres. The images are from the vegetal pole (top two rows) and the animal pole (bottom row).

(B) The relative numbers of cells rescued by the indicated regulators is shown as the rescue index. Each transfection was divided, and half the cells were plated in the presence of Tc and the rest in its absence. The rescue index was calculated by dividing the number of alkaline phosphatase (AP)-positive colonies obtained in the absence and presence of Oct4 (± Tc). Data represent the mean values obtained from three independent experiments.

(C–E) Morphology of ESC colonies supported by wild-type PouV proteins (C), activator fusions (D), and repressor fusions (E). The colonies were generated by stable transfection of the ZHBTc4 ESCs with vectors expressing one or another wild-type PouV or PouV fusion proteins and the *Pac* gene (which renders cells puromycin resistant) from an internal ribosome entry site (IRES) in the same message, all under the control of the strong CAG promoter. Constitutive expression of the exogenous fusion proteins in the absence of Oct4 was selected on the basis of Puromycin resistance in the presence of Tc. Cultures were stained for AP (red).

plus either wild-type human OCT4 or OCT4 fusion proteins (Figure S3) (Papapetrou et al., 2009). Figure 2B shows that three OCT4-repressing fusions eliminated iPSC formation and that two activating fusions labeled "weak" and "medium" (see Table 1 legend) substituted efficiently for wild-type OCT4. The activator fusion labeled "strong" worked somewhat less efficiently than did its weaker counterparts, but Figure 2C shows that decreasing its concentration improved its activity in this assay. Thus, Oct4-activator fusions, but not -repressor fusions, promote iPSC formation.

Gene Expression in Activator-Induced and -Maintained Pluripotent Cell Lines

Murine ESC Lines

We used microarrays to compare the transcriptional profiles of ESCs maintained, respectively, by wild-type Oct4 and by Oct4λVP2. A set of genes directly or indirectly responsive to wild-type Oct4 was previously determined by measuring changes in gene expression after knockdown of endogenous Oct4 (by adding Tc to the parental ZHBTc4 ESCs) (Sharov et al., 2008). Putative direct targets of Oct4, from among these genes, were identified as being rapidly responsive and having at least one Oct4 binding site in the vicinity of the transcription start site on the basis of the ChIP-PET method. Of the 372 putative Oct4-dependent targets (from Sharov et al., 2008), some 94% were expressed at normal or higher levels in Oct4λVP2- maintained cells. Expression of the remaining 6% (23 genes) was reduced more than 2-fold

Cell PRESS
Open ACCESS

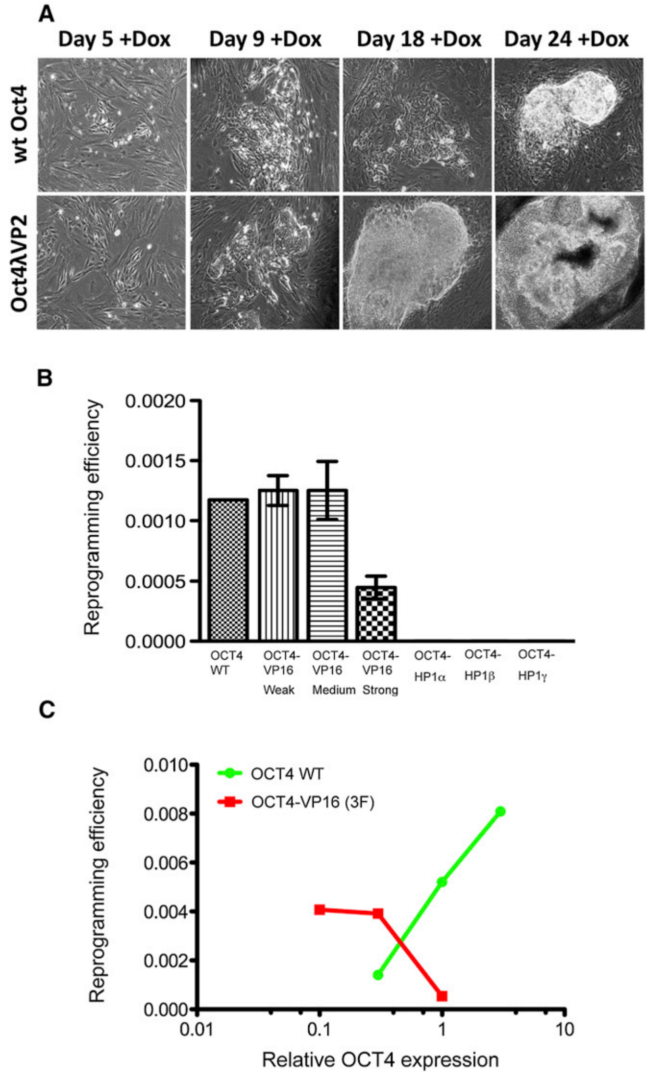

Figure 2. Oct4-Activator Fusions Support iPSC Generation

(A) Morphology of colonies resulting from mouse embryonic fibroblasts (MEF) transfection with either wild-type Oct4 or Oct4λVP2 alongside the other reprogramming factors (cMyc, Klf4, and Sox2) at days 5, 9, 18, and 24 after transfection in the presence of the Tc analog Dox. MEFs were transfected with a *piggy*Bac transposon bearing a single-message multigene expression cassette under the control of a Tc-inducible promoter.

(B) OCT4 activator fusions support human iPSC generation. MRC-5 human embryonic fibroblasts were transduced with combinations of four lentiviral bicistronic vectors coexpressing each reprogramming factor, linked by a P2A peptide, to a fluorescent protein (Papapetrou et al., 2009): SOX2 together with mCitrine, KLF4 with mCherry, cMYC with mCerulean, and either wild-type OCT4 or OCT4 fusion proteins with vexGFP. The efficiency was calculated with the use of the experimental setup depicted in Figure S3C. In brief, the number of Tra-1-81^{+} colonies (scored by immunostaining on day 15 after transduction) per number of plated cells divided by the fraction of quadruple positive cells as estimated by flow cytometry on day 2 after transduction.

(C) Lower levels of the strong activator (OCT4-VP16 [3F]) work more efficiently in iPSC formation. MRC-5 human embryonic fibroblasts were transduced as in (B) with the use of 3-fold titrations of the vector expressing either wild-type OCT4 or OCT4-VP16 (3F) and constant amounts of the other three vectors (SOX2, KLF4, cMYC). The reprogramming efficiency was calculated as in (B). See also Figure S3C and its associated legend.

(Figure 3A). Among these 23 there were only two genes with known pluripotency-related activity: *Sall4* and *Lefty1*. Most genes associated with differentiation (e.g., *Cdx2*, *Eomes*, *Fgf5*, *Gata6*, *Gata3*, *Igf2*, *Hand1*, *Esx1*) were either reduced or expressed at normal levels (Table S1). All other pluripotency-related genes were expressed at either normal or higher levels in Oct4λVP2-rescued cells (Table S1, Figure 3B; also see Figure S7), with a small set of primordial germ cell and pluripotency markers expressed at levels greater than 2-fold above wild-type cells, including *Gdf3*, *Dppa2*, *Dppa3* (*Stella*), *Eras*, *Tcfcp2l1*, *Id3*, *Fbxo15*, *Sox15*, and *Nanog*. Nanog was expressed at an average of 2- to 5-fold higher than in ordinary ESCs, and its distribution was more homogeneous (Figure S4E). Gene ontology (GO) annotations of the upregulated and downregulated genes in the Oct4λVP2 cell line with more than 2-fold changes are listed in Table S2. While there does not appear to be a single biological process common to the upregulated genes in Oct4λVP2 cells, the downregulated gene set was enriched in functions associated with embryonic development and differentiation. These GO terms include a list of developmental transcription factors, cell differentiation, system development, epithelial differentiation, sex differentiation, cell migration, Wnt signaling, and several categories related to neural development.

Murine and Human iPSC Lines

Endogenous pluripotency genes, including *Oct4*, *Klf4*, *Sox2*, and *c-Myc*, were expressed in murine iPSCs generated from fibroblasts with Oct4λVP2 (plus the other three factors) (Figure 3C, Figure S5). Expression of Nanog and Dppa4 at early times after transduction of human fibroblasts with OCT4 fusions was increased by increasingly stronger versions of OCT4-VP16 (Figure 3D). These markers were only modestly induced at this early time point by wild-type OCT4, and not at all by OCT4-HP1.

ESCs Rescued and Maintained by Activator Fusions Are Resistant to Differentiation

Overexpression of Nanog has been reported to counter the induction of differentiation as a result of LIF removal (Chambers et al., 2003). Consistent with these findings, our Oct4λVP2-supported murine ESCs, which overexpress Nanog, are able to grow in the absence of LIF, as shown in Figure 4A. Oct4λVP2-maintained ESC lines were AP positive and exhibited undifferentiated ESC morphology when grown with or without LIF. These cells did, however, grow more slowly in the absence of LIF than in its presence (Figure 4 and Figures S6B and S6C). To better understand the response of Oct4λVP2-supported cells to LIF, we compared mRNA from Oct4λVP2-rescued cell lines and two sets of control lines (Oct4-rescued and the parental ZHBTc4 cells) in response to LIF withdrawal (Figures 4B and 4C, Table S3, and Figure S6). LIF withdrawal caused similar changes in gene expression in the latter two lines, with a single set of genes decreased (cluster a, Figure 4B) and a second set increased (cluster b, Figure 4B). However, LIF withdrawal from Oct4λVP2-maintained cell lines caused little change in expression of genes in either set. For example, expression of the pluripotency genes *Nanog*, *Klf4*, *Sox2*, and *Rex1* was downregulated in the control cells after LIF withdrawal, but their expression was unaffected in Oct4λVP2-maintained cells. The endodermal markers Gata4 and Gata6, upregulated upon LIF withdrawal

Cell PRESS
Open ACCESS

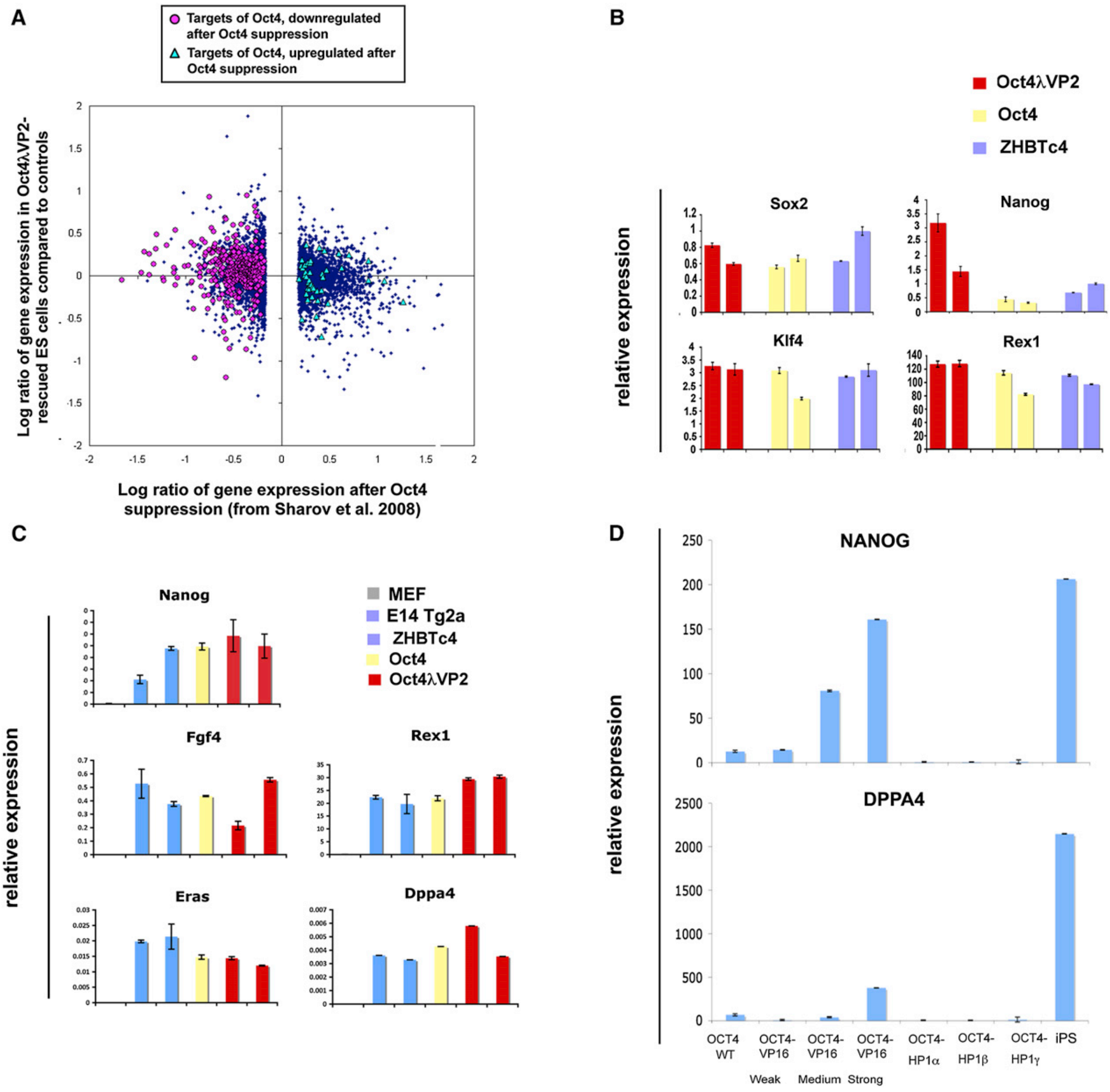

Figure 3. Oct4 Activator Fusions Induce and Maintain the Expression of Pluripotency-Related Genes

(A) Oct4λVP2 enhances the expression of Oct4-positively-regulated genes and inhibits the expression of those genes negatively regulated by Oct4. We compared the total set of genes changing when Oct4λVP2-supported cells were compared to controls, to the complete set of genes responsive to Oct4. The majority of these genes (94%) were expressed at normal (around the *x* axis) or higher than normal (above the *x* axis) levels in Oct4λVP2 cells. The basis for this plot was the difference between the log expression of genes in Oct4λVP2 cells and the average log expression in both controls, plotted against the maximum gene expression change (log ratio) in a time course after *Oct4-Tc* silencing (addition of Tc) in ZHBTc4 ESCs (the time course data from Sharov et al., 2008). Genes with < 1.5-fold change of expression after *Oct4-Tc* silencing are not shown. Oct4-dependent genes that also contain Oct4, Nanog, and Sox2 binding sites are indicated as targets (light blue or purple; see Sharov et al., 2008).

(B) Expression of pluripotency genes in Oct4λVP2-rescued cells. Expression levels of pluripotency-related genes including *Nanog*, *Sox2*, *Klf4*, and *Rex1* were determined by qRT-PCR. Expression levels of all analyzed genes were normalized to *TBP* levels for each sample.

(C) iPSCs generated with the use of Oct4λVP2 express levels of pluripotency markers similar to those of normal iPSCs. Expression levels of a range of pluripotent cell markers were assessed by qRT-PCR. *Nanog, Fgf4*, *Rex1*, *Dppa4*, and *Eras* expression is shown in wild-type Oct4 and Oct4λVP2 iPSCs. Duplicate bars represent independent iPSC lines. Expression levels were normalized to *TBP* levels for each sample. Key indicates the Oct4 protein used for reprogramming. E14 (embryonic day 14) Tg2A ESCs and MEFs were used for the sake of comparison.

(D) Wild-type OCT4 and its activator fusions, but not OCT4-HP1 repressor fusions, induce immediate early expression of pluripotency markers, *NANOG* and *DPPA4*. MRC5 human embryonic fibroblast cells were harvested 48 hr after viral transduction for mRNA analysis. Expression levels in an iPSC line generated with the use of wild-type forms of all four factors are shown for comparison. Levels of gene expression are relative to RPL7 and normalized to nontransduced control.

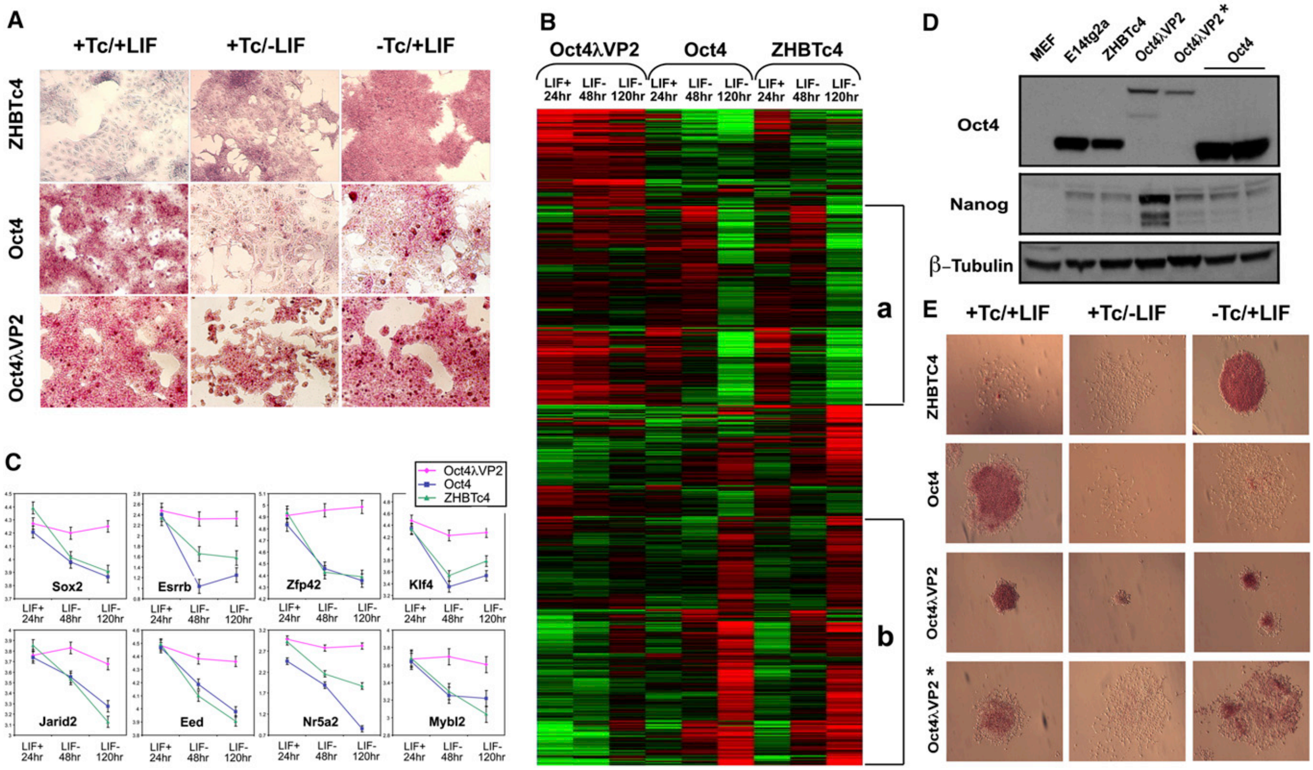

Figure 4. Differential Effect of Oct4λVP2 Dose on ESC Differentiation

(A) Oct4λVP2 maintains undifferentiated phenotypes irrespective of the presence of LIF or additional Oct4. Oct4λVP2-supported and control ESC lines were plated in the absence of LIF or absence of Tc (Oct4 overexpression) for 5 days. Cultures were stained for AP (red).

(B) Changes in gene expression normally associated with LIF withdrawal do not occur in Oct4λVP2-supported cells. Here, a heat map shows the 3,000 genes with the most significant differences in gene expression after LIF withdrawal. Two clusters are bracketed by “a” and “b”: a, genes that are downregulated in Oct4 and ZHBTc4 cells, but not in Oct4λVP2 cells, after 120 hr of LIF withdrawal; b, genes that are upregulated in Oct4 and ZHBTc4 cells, but not in Oct4λVP2 cells, after 120 hr of LIF withdrawal.

(C) Pluripotency genes are supported in the absence of LIF in Oct4λVP2 cells. Shown are plots comparing mean log intensity values of representative pluripotency genes. Error bars represent SD between expression levels in two independent clones.

(D) Oct4λVP2* cells compared to original Oct4λVP2 and control cell lines. These cells, generated in reduced puromycin, express reduced levels of Oct4λVP2 and Nanog. Western blots comparing expression levels of Oct4 and Nanog in the different rescued cell lines are shown.

(E) Oct4λVP2* cell lines differentiate in response to LIF withdrawal and Oct4 overexpression. Cells were plated at clonal density in the absence of LIF or Oct4 overexpression for 5 days. Two clones for each cell line except ZHBTc4 were used and stained for AP activity (red). Representative colonies are shown for each condition.

from typical ESCs, remained unaffected by LIF withdrawal from Oct4λVP2-supported ESCs. Expression of certain mesoderm markers that are normally expressed alongside Oct4 in the primitive streak (e.g., Brachyury and Wnt 3) were increased after LIF removal from Oct4λVP2-supported cells (Figure 4C and Figure S7). When examined globally, there was a striking negative correlation between the set of genes that normally respond to LIF and those expressed at high levels in Oct4λVP2-maintained cells grown in the absence of LIF (comparison based on average log ratio, $r = -0.695$, $t = 153$, $p < 0.0001$; also see Figure S6G).

Despite the robust expression of *Nanog* and other pluripotency-related genes in Oct4λVP2-maintained ESCs, and the conferred LIF independence just described, the expression of Oct4λVP2 itself was 2- to 5-fold lower than that of Oct4 in ESCs maintained by wild type Oct4 (Figure 4D, Figure S4E). We repeated the original Oct4λVP2-rescue experiment to generate new stable cell lines (see Figure S4A), adding the same amount of Tc to the medium but a reduced amount of puromycin. This reduced stringency of selection enabled the isolation of new Oct4λVP2-supported cell lines (Oct4λVP2*), which expressed even lower levels (from 1.4- to 2-fold mRNA and/or protein) of Oct4λVP2 (Figure 4D). These lines also express correspondingly lower levels of Nanog and other pluripotency factors, similar to levels found in ESCs and in cells rescued by Oct4 (Figure 4D, Figures S8C and S8D). Upon removal of LIF, these cells differentiated to extents similar to those of wild-type Oct4-rescued ESCs and parental ZHBTc4 control cell lines (Figure 4E). Colonies generated from clonal growth of Oct4λVP2* cell lines appeared similar to wild-type Oct4-supported cell lines, both forming differentiated cells at the periphery (Figures S8A and S8B) and both sensitive to the differentiation-induced effect of Oct4 overexpression (Figure 4E).

DISCUSSION

Our findings support the idea that the necessary and sufficient action of Oct4 in inducing iPSC formation, and in blocking differentiation in vitro and in vivo, is to directly activate (rather than directly repress) expression of specific target genes. Thus, in both mouse ESCs and *Xenopus* embryos, PouV/Oct4-repressor fusion proteins induced differentiation, whereas the activator forms maintained the undifferentiated pluripotent state. Our results are consistent with a "positive only" model (based on a different line of experimentation) for maintenance of pluripotency of ESCs (Chamberlain et al., 2008).

Moreover, the Oct4-activator fusions, but not the -repressor fusions (along with additional transcription factors), induced iPSC formation. Analysis of gene expression profiles revealed that Oct4-activator fusions specifically enhanced expression of many genes identified as required for pluripotency, but not of genes that drive differentiation. One of our ESC lines (see below) maintains its ESC state even after withdrawal of LIF. These cells do not differentiate in response to additional Oct4 and thus do not display the usual Oct4 overexpression phenotype. These results are consistent with recent reports that claim enhanced efficiency in reprogramming as a result of the use of Oct4-VP16, Sox2-VP16, and Nanog-VP16 fusion proteins (Wang et al., 2011a) or Oct4-MyoD (Hirai et al., 2011).

We cannot exclude the possibility that Oct4 can repress certain genes, particularly in cases where it may bind cooperatively with other pluripotency factors with repression activity, and this might also explain its association with corepressor complexes. Such an activity, were it to exist, would not undermine the central finding of this paper, namely that activation and not repression is sufficient to induce and maintain the undifferentiated state. It is highly unlikely that our Oct4-VP16 fusion can repress certain genes as it activates others. The fusion activates two reporters bearing two different Oct4 DNA-recognition elements, both derived from natural enhancer elements. Moreover, we found that the activator fusion activated transcription of *Xist*, putatively a gene repressed by Oct4 in vivo (Navarro et al., 2008) (Table S1). These and other observations indicate that the fusion protein Oct4λVP2 is likely an activator of all Oct4 targets, or at least of all those required for pluripotency.

Our work contrasts with the suggestion of Loh and Lim (2011) that, in maintaining the ESC state, Oct4 attempts to drive differentiation along a specific path (see Introduction). It would also contradict the notion that an essential role of Oct4 in maintaining pluripotency is to directly repress genes required for differentiation (Bilodeau et al., 2009; Boyer et al., 2005; Boyer et al., 2006; Chen et al., 2008; Kim et al., 2008; Loh et al., 2006; Pasini et al., 2010). It would, however, be consistent with the suggestion of Ying et al. (2008) that pluripotency is a "ground state" maintained by factors that prevent differentiation in response to (for example) paracrine signaling. Genes activated by Oct4 (and perhaps other pluripotency factors) would perform this function. This picture would cast pluripotency as another example of a "differentiated" state maintained by positive feedback. Thus, for other cases of terminal differentiation, an activator (or groups of self-reinforcing activators) maintains expression of its own gene as it activates expression of genes required for the differentiated state (Hobert, 2008). Oct4 is widely believed to maintain its own expression in a feedback loop involving, perhaps, other pluripotency gene regulators (Chew et al., 2005; Hall et al., 2009; Sharov et al., 2008). We found that increasing the activating region strength of the Oct4 fusions (bearing VP16 derivatives), as well as increasing the concentration of one of them, resulted in higher levels of Nanog expression, suggesting that Oct4 directly activates Nanog as part of this loop.

How, then, would we rationalize this proposal with findings and models of others? First, we note the importance attributed by Loh and Lim (2011) to the finding that overexpression of Oct4 causes differentiation, a result taken to imply that Oct4 activates genes required for differentiation, not for pluripotency. But overexpression experiments can be difficult to interpret. An activator works in a concentration-dependent manner, binding to proper DNA sites (usually with other proteins) over a certain concentration range and then, at higher concentrations, binding less specifically to DNA. And, by virtue of protein-protein interactions, an overexpressed activator can inhibit (squelch) the effect of other activators and even its own action (Gill and Ptashne, 1988; Ptashne, 2009). Indeed, it has been reported that overexpression of Oct4 lacking a functional DNA-binding domain induces ESCs to differentiate (Niwa et al., 2002), a result indicating that the phenotype is not directly attributable to the ordinary action of Oct4.

In our experiments, any side effects of overexpression evidently have been eliminated by the use of the strong activator fusions. Thus, in a line of ESCs maintained by Oct4λVP2, the level of the Oct4λVP2 protein is low, significantly lower than that of wild-type Oct4 found in the typical ESC. These cells express high levels of select pluripotency factors, including Nanog, and are resistant to the ordinary inducing effect of differentiation upon LIF removal. These cells also, unlike ESCs maintained by exogenously added Oct4, do not differentiate when additional Oct4 is expressed. Another line, selected to express even lower levels of the activator, behaves like a typical ESC, subject to differentiation upon LIF removal, despite the very low level of expression of the activator fusion, and this line differentiates upon expression of additional Oct4.

Second, we note the importance given to genome-wide ChIP experiments showing that, in ESCs, Oct4 can be found bound to regulatory elements at certain genes that are not expressed—these genes become expressed upon differentiation. But lacking further analysis, these purported facts do not show that Oct4 is working as a repressor at those genes. Perhaps Oct4 is bound to genes in a nonfunctional state, awaiting relief from, or addition of, some partner to work. Polycomb, for example, is found at many silent genes in ESCs, but its removal has little effect on the maintenance of pluripotent gene expression (Schoeftner et al., 2006), and there are indications that polycomb remains at these genes during very early steps in differentiation (J.M.B. and W.A. Bickmore, unpublished data). There may be a misleading assumption at work here, namely that for a gene to be "off" it must be actively repressed. Though this is true for most genes in bacteria, it is not true in general for genes in eukaryotes. In bacteria, specific repressors are required to eliminate an otherwise significant basal level expression upon

removal of activators, but this is evidently not true for eukaryotic genes. Rather, it seems, the general inhibiting effect of nucleosomes maintains basal expression at very low levels (Struhl, 1999; Wang et al., 2011b). Thus, without activators that would turn on the differentiation genes, there would be no need for repressors to turn them off. And so in ESCs, where activators maintain expression of pluripotency genes, differentiation genes could simply be off (by default, as it were) until activated by some activators that are rendered functional by virtue of gain (or loss) of a signal.

EXPERIMENTAL PROCEDURES

ESCs were cultured on 0.1% gelatin-coated flasks or plates (IWAKI) in Glasgow modified Eagle's medium (GIBCO) containing nonessential amino acids, glutamine, sodium pyruvate, 0.1 mM mercaptoethanol, 10% fetal calf serum (FCS), and LIF as described previously (Livigni et al., 2009; Morrison and Brickman, 2006). LIF withdrawal and Oct4 overexpression was performed according to Morrison and Brickman (2006). For ESC self-renewal rescue experiments, 2 × 10^7 ZHBTc4 ESCs were electroporated with 100 μg of linearized plasmid DNA followed by culture with or without 2 μg/ml Tc (Sigma) for 2 days. Cells were then cultured in 2 μg/ml puromycin (Sigma) with or without Tc for 7 days. The resulting colonies were stained for alkaline phosphatase (AP) activity (Sigma-Aldrich) or expanded as clonal lines (Morrison and Brickman, 2006). The cDNAs used for ESC rescue were inserted into pCAGIP vector (Niwa et al., 2002; Niwa et al., 1991).

Luciferase assays were performed as described previously (Brickman et al., 2001; Morrison and Brickman, 2006). ZHBTc4 ESCs (1 × 10^5) were plated on a 24-well plate with 2 μg/ml of Tc. Twenty-four hours after plating, the reporter and test plasmid were transfected with the use of Lipofactamine 2000 (Invitrogen). Twenty-four to forty-eight hours after transfection, the cells were collected and lysed, and luciferase assay was performed with the Dual-Luciferase Reporter Assay System (Promega). Reporter plasmids were a kind gift from Professor Hitoshi Niwa (Riken Institute, Japan) (Niwa et al., 2002). The expression vector used was the pCAG-IP plasmid.

Growth rates were assessed in 96-well plates with CellTiter 96 Aqueous One Solution Cell Proliferation Assay (Promega). A total of 1,000 cells were plated per well and assayed every 24 hr according to the manufacturer's instructions. AP staining was performed with the Leukocyte Alkaline Phosphatase Assay Kit (Sigma).

For antibody staining, cells were washed twice in PBS, followed by fixation in 4% paraformaldehyde. Cells were then permeabilized in PBS + 0.1% Tween 20 (PBST), and blocking was performed by adding 1% bovine serum albumin (BSA) (Sigma) PBST to the cells for 30 min at room temperature. Primary antibodies (see Table S4 for details) were incubated overnight at 4°C, followed by three washes in PBST for 10 min each. Alexa Fluor-conjugated secondary antibodies (see Table S4 for details) were diluted in blocking solution and added to the cells for 3 hr at room temperature with the DAPI solution. Finally, cells were washed three times in PBST.

Flow cytometry was used to analyze the percentage of ESC clones expressing the cell-surface markers SSEA1 and E-Cadherin (for the antibodies, see Table S4). Cells were collected into the cell-dissociation buffer (GIBCO) and incubated at 37°C for 10 min. Single-cell suspension was achieved by gentle repeated pipetting. After PBS washes, cells were resuspended in 500 μl FACS buffer (1× PBS and 10% FCS). To mark apoptotic cells, 5 μl/10^6 cells of 7AAD solution (BD PharMingen) was added to cells. Samples were analyzed in a FACSCalibur flow cytometer (BD Biosciences). Data were analyzed with the Cell Quest software (BD Biosciences).

For qRT-PCR analysis, total RNA was prepared from cells with the use of Trizol reagent (Invitrogen). RNA (0.5–1 μg) was used as a template for cDNA synthesis with the use of Superscript III (Invitrogen). qRT-PCR was performed with the use of a LightCycler 480 (Roche). Primers and PCR conditions are listed in Table S5.

Embryo injection, culture, and in situ hybridization were described in Zamparini et al., 2006 and Morrison and Brickman, 2006. To generate RNA for injections, pCS2+ plasmids harboring the target cDNAs *Xlpou91λVP2*, *Oct4λVP2*, *Xlpou91λEnR*, *Oct4λEnR*, *Oct4*, and *Xlpou91* were linearized with *BssHI*I and used as a template for RNA synthesis with SP6 polymerase (Promega). Total RNA was prepared from five embryos with the RNeasy Mini Kit (QIAGEN) and DNase1 treatment. RNA (500 ng) was used as a template for cDNA synthesis with Superscript III (QIAGEN). Real-time PCR was carried out with a Lightcycler 480 (Roche). Real-time PCR primers are listed in Table S5.

For microarray analysis, two independent clones from each cell line were used as biological replications. Cy3-CTP-labeled sample targets were prepared from 2.5 μg of total RNA with the use of a Low RNA Input Fluorescent Linear Amplification Kit (Agilent). Cy5-CTP-labeled reference target was produced from 2.5 μg of Stratagene Universal Mouse Reference RNA. Purified target RNAs were hybridized to the NIA Mouse 44K Microarray version 3.0 (whole-genome 60-mer oligo arrays, Agilent Technology, design ID 015087) (Carter et al., 2005) according to the manufacturer's protocol (Two-Color Microarray-Based Gene Expression Analysis Protocol, version 5.0.1). Data were analyzed via NIA Array Analysis (Sharov et al., 2005) under standard statistical conditions (FDR < 0.05, 2-fold expression level change).

For mouse iPSC generation, *Piggy*Bac transposons containing *Oct4* and *Oct4λVP2* were constructed as described previously (Kaji et al., 2009). Mouse embryonic fibroblasts (MEFs) were isolated from 13.5 dpc (days postcoitus) ROSA26 knockin rtTA-IRES-GFP embryos. MEF Nucleofector Kit 2 (Amaxa) and program T-20 were used for nucleofection. A total of 2 × 10^6 cells were transfected, and 5 × 10^5 cells were seeded on gelatine in 10 cm^2 dishes. Cells were cultured in ESC media supplemented with 1.5 ug of Dox. Colonies arising after transfection were picked at day 24, trypsinized, and replated on gelatine. Dox was removed from the culture after several passages.

Lentiviral vector production, human iPSC generation, and immunostaining were performed as previously described (Papapetrou et al., 2009). Separate lentiviral constructs encoding full-length human Oct4, Sox2, Klf4, and cMyc linked by a P2A peptide to vexGFP, mCitrine, mCherry, and mCerulean, respectively, were used. MRC-5 human embryonic fibroblasts (American Type Culture Collection [ATCC]) were transduced with supernatants of the four lentiviral vectors in the presence of 4 μg/ml polybrene for approximately 16 hr. Three days after transduction, the cells were plated at a density of 20,000 cells per 60 mm dish on a feeder layer of mitomycin-C-treated MEFs. Analysis of the four vector-encoded transcription factors' expression by flow cytometry 3 days after transduction was used to identify groups with similar expression levels (mean fluorescence intensity) for comparison. The next day, the medium was switched to hESC medium supplemented with 6 ng/mL FGF2 and was replaced every day thereafter. Then, 16-18 days after transduction, colonies with hESC morphology were mechanically dissociated and transferred into 24-well plates on MEFs. Cells were thereafter passaged with dispase and expanded to establish hiPSC lines.

ACCESSION NUMBERS

The GEO accession number for the microarray data reported in this paper is GSE24563.

SUPPLEMENTAL INFORMATION

Supplemental Information includes eight figures and five tables and can be found with this article online at doi:10.1016/j.celrep.2011.12.002.

LICENSING INFORMATION

This is an open-access article distributed under the terms of the Creative Commons Attribution-Noncommercial-No Derivative Works 3.0 Unported License (CC-BY-NC-ND; http://creativecommons.org/licenses/by-nc-nd/3.0/legalcode).

ACKNOWLEDGMENTS

We would like to thank Hitoshi Niwa and Austin Smith for provision of materials; Laxmi M.S. Tirunagari for technical assistance; Michel Sadelain for advice; and

Alfonso Martinez Arias, Sally Lowell, Val Wilson, and the entire Brickman and Ptashne labs for critical reading of this manuscript. This work was supported by the BBSRC (BB/C506605), Scottish Funding Council, the Medical Research Council (MRC G0701428) (to J.M.B.), the Intramural Research Program of the NIH, the National Institute on Aging (Z01AG AG000656, Z01AG000662) (to M.S.H.K.), and the New York Stem Cell Science (NYSTEM) Program (C026407) (to M.P.). F.H. was supported by a scholarship from the Algerian Government. J.M.B. is an MRC Senior Non-Clinical Research Fellow.

Received: October 17, 2011
Revised: November 19, 2011
Accepted: December 15, 2011
Published online: February 16, 2012

REFERENCES

Ang, Y.S., Tsai, S.Y., Lee, D.F., Monk, J., Su, J., Ratnakumar, K., Ding, J., Ge, Y., Darr, H., Chang, B., et al. (2011). Wdr5 mediates self-renewal and reprogramming via the embryonic stem cell core transcriptional network. Cell *145*, 183–197.

Baron, U., Gossen, M., and Bujard, H. (1997). Tetracycline-controlled transcription in eukaryotes: novel transactivators with graded transactivation potential. Nucleic Acids Res. *25*, 2723–2729.

Bilodeau, S., Kagey, M.H., Frampton, G.M., Rahl, P.B., and Young, R.A. (2009). SetDB1 contributes to repression of genes encoding developmental regulators and maintenance of ES cell state. Genes Dev. *23*, 2484–2489.

Boyer, L.A., Lee, T.I., Cole, M.F., Johnstone, S.E., Levine, S.S., Zucker, J.P., Guenther, M.G., Kumar, R.M., Murray, H.L., Jenner, R.G., et al. (2005). Core transcriptional regulatory circuitry in human embryonic stem cells. Cell *122*, 947–956.

Boyer, L.A., Plath, K., Zeitlinger, J., Brambrink, T., Medeiros, L.A., Lee, T.I., Levine, S.S., Wernig, M., Tajonar, A., Ray, M.K., et al. (2006). Polycomb complexes repress developmental regulators in murine embryonic stem cells. Nature *441*, 349–353.

Brickman, J.M., Jones, C.M., Clements, M., Smith, J.C., and Beddington, R.S.P. (2000). Hex is a transcriptional repressor that contributes to anterior identity and suppresses Spemann organiser function. Development *127*, 2303–2315.

Brickman, J.M., Clements, M., Tyrell, R., McNay, D., Woods, K., Warner, J., Stewart, A., Beddington, R.S., and Dattani, M. (2001). Molecular effects of novel mutations in Hesx1/HESX1 associated with human pituitary disorders. Development *128*, 5189–5199.

Cao, Y., Siegel, D., and Knöchel, W. (2006). Xenopus POU factors of subclass V inhibit activin/nodal signaling during gastrulation. Mech. Dev. *123*, 614–625.

Carter, M.G., Sharov, A.A., VanBuren, V., Dudekula, D.B., Carmack, C.E., Nelson, C., and Ko, M.S. (2005). Transcript copy number estimation using a mouse whole-genome oligonucleotide microarray. Genome Biol. *6*, R61.

Chamberlain, S.J., Yee, D., and Magnuson, T. (2008). Polycomb repressive complex 2 is dispensable for maintenance of embryonic stem cell pluripotency. Stem Cells *26*, 1496–1505.

Chambers, I., Colby, D., Robertson, M., Nichols, J., Lee, S., Tweedie, S., and Smith, A. (2003). Functional expression cloning of Nanog, a pluripotency sustaining factor in embryonic stem cells. Cell *113*, 643–655.

Chen, X., Xu, H., Yuan, P., Fang, F., Huss, M., Vega, V.B., Wong, E., Orlov, Y.L., Zhang, W., Jiang, J., et al. (2008). Integration of external signaling pathways with the core transcriptional network in embryonic stem cells. Cell *133*, 1106–1117.

Chew, J.L., Loh, Y.H., Zhang, W., Chen, X., Tam, W.L., Yeap, L.S., Li, P., Ang, Y.S., Lim, B., Robson, P., and Ng, H.H. (2005). Reciprocal transcriptional regulation of Pou5f1 and Sox2 via the Oct4/Sox2 complex in embryonic stem cells. Mol. Cell. Biol. *25*, 6031–6046.

Evans, M.J., and Kaufman, M.H. (1981). Establishment in culture of pluripotential cells from mouse embryos. Nature *292*, 154–156.

Gill, G., and Ptashne, M. (1988). Negative effect of the transcriptional activator GAL4. Nature *334*, 721–724.

Hall, J., Guo, G., Wray, J., Eyres, I., Nichols, J., Grotewold, L., Morfopoulou, S., Humphreys, P., Mansfield, W., Walker, R., et al. (2009). Oct4 and LIF/Stat3 additively induce Krüppel factors to sustain embryonic stem cell self-renewal. Cell Stem Cell *5*, 597–609.

Hay, D.C., Sutherland, L., Clark, J., and Burdon, T. (2004). Oct-4 knockdown induces similar patterns of endoderm and trophoblast differentiation markers in human and mouse embryonic stem cells. Stem Cells *22*, 225–235.

Hirai, H., Tani, T., Katoku-Kikyo, N., Kellner, S., Karian, P., Firpo, M., and Kikyo, N. (2011). Radical acceleration of nuclear reprogramming by chromatin remodeling with the transactivation domain of MyoD. Stem Cells *29*, 1349–1361.

Hobert, O. (2008). Regulatory logic of neuronal diversity: terminal selector genes and selector motifs. Proc. Natl. Acad. Sci. USA *105*, 20067–20071.

Kaji, K., Norrby, K., Paca, A., Mileikovsky, M., Mohseni, P., and Woltjen, K. (2009). Virus-free induction of pluripotency and subsequent excision of reprogramming factors. Nature *458*, 771–775.

Kim, J., Chu, J., Shen, X., Wang, J., and Orkin, S.H. (2008). An extended transcriptional network for pluripotency of embryonic stem cells. Cell *132*, 1049–1061.

Kim, J.B., Sebastiano, V., Wu, G., Araúzo-Bravo, M.J., Sasse, P., Gentile, L., Ko, K., Ruau, D., Ehrich, M., van den Boom, D., et al. (2009). Oct4-induced pluripotency in adult neural stem cells. Cell *136*, 411–419.

Lavial, F., Acloque, H., Bertocchini, F., Macleod, D.J., Boast, S., Bachelard, E., Montillet, G., Thenot, S., Sang, H.M., Stern, C.D., et al. (2007). The Oct4 homologue PouV and Nanog regulate pluripotency in chicken embryonic stem cells. Development *134*, 3549–3563.

Livigni, A., Villegas, S.N., Oikonomopoulou, I., Rahman, A., Morrison, G.M., and Brickman, J.M. (2009). Differentiation of embryonic stem cells into anterior definitive endoderm. Curr. Protoc. Stem Cell Biol. *Chapter 1*, 1G, 3.

Loh, K.M., and Lim, B. (2011). A precarious balance: pluripotency factors as lineage specifiers. Cell Stem Cell *8*, 363–369.

Loh, Y.H., Wu, Q., Chew, J.L., Vega, V.B., Zhang, W., Chen, X., Bourque, G., George, J., Leong, B., Liu, J., et al. (2006). The Oct4 and Nanog transcription network regulates pluripotency in mouse embryonic stem cells. Nat. Genet. *38*, 431–440.

Lunde, K., Belting, H.G., and Driever, W. (2004). Zebrafish pou5f1/pou2, homolog of mammalian Oct4, functions in the endoderm specification cascade. Curr. Biol. *14*, 48–55.

Martin, G.R. (1981). Isolation of a pluripotent cell line from early mouse embryos cultured in medium conditioned by teratocarcinoma stem cells. Proc. Natl. Acad. Sci. USA *78*, 7634–7638.

Morrison, G.M., and Brickman, J.M. (2006). Conserved roles for Oct4 homologues in maintaining multipotency during early vertebrate development. Development *133*, 2011–2022.

Navarro, P., Chambers, I., Karwacki-Neisius, V., Chureau, C., Morey, C., Rougeulle, C., and Avner, P. (2008). Molecular coupling of Xist regulation and pluripotency. Science *321*, 1693–1695.

Nichols, J., Zevnik, B., Anastassiadis, K., Niwa, H., Klewe-Nebenius, D., Chambers, I., Schöler, H., and Smith, A. (1998). Formation of pluripotent stem cells in the mammalian embryo depends on the POU transcription factor Oct4. Cell *95*, 379–391.

Niwa, H., Yamamura, K., and Miyazaki, J. (1991). Efficient selection for high-expression transfectants with a novel eukaryotic vector. Gene *108*, 193–199.

Niwa, H., Burdon, T., Chambers, I., and Smith, A. (1998). Self-renewal of pluripotent embryonic stem cells is mediated via activation of STAT3. Genes Dev. *12*, 2048–2060.

Niwa, H., Miyazaki, J., and Smith, A.G. (2000). Quantitative expression of Oct-3/4 defines differentiation, dedifferentiation or self-renewal of ES cells. Nat. Genet. *24*, 372–376.

Niwa, H., Masui, S., Chambers, I., Smith, A.G., and Miyazaki, J. (2002). Phenotypic complementation establishes requirements for specific POU domain and

generic transactivation function of Oct-3/4 in embryonic stem cells. Mol. Cell. Biol. *22*, 1526–1536.

Ohashi, Y., Brickman, J.M., Furman, E., Middleton, B., and Carey, M. (1994). Modulating the potency of an activator in a yeast in vitro transcription system. Mol. Cell. Biol. *14*, 2731–2739.

Palmieri, S.L., Peter, W., Hess, H., and Schöler, H.R. (1994). Oct-4 transcription factor is differentially expressed in the mouse embryo during establishment of the first two extraembryonic cell lineages involved in implantation. Dev. Biol. *166*, 259–267.

Pan, G.J., Chang, Z.Y., Schöler, H.R., and Pei, D. (2002). Stem cell pluripotency and transcription factor Oct4. Cell Res. *12*, 321–329.

Papapetrou, E.P., Tomishima, M.J., Chambers, S.M., Mica, Y., Reed, E., Menon, J., Tabar, V., Mo, Q., Studer, L., and Sadelain, M. (2009). Stoichiometric and temporal requirements of Oct4, Sox2, Klf4, and c-Myc expression for efficient human iPSC induction and differentiation. Proc. Natl. Acad. Sci. USA *106*, 12759–12764.

Pardo, M., Lang, B., Yu, L., Prosser, H., Bradley, A., Babu, M.M., and Choudhary, J. (2010). An expanded Oct4 interaction network: implications for stem cell biology, development, and disease. Cell Stem Cell *6*, 382–395.

Pasini, D., Cloos, P.A., Walfridsson, J., Olsson, L., Bukowski, J.P., Johansen, J.V., Bak, M., Tommerup, N., Rappsilber, J., and Helin, K. (2010). JARID2 regulates binding of the Polycomb repressive complex 2 to target genes in ES cells. Nature *464*, 306–310.

Ptashne, M. (2009). Binding reactions: epigenetic switches, signal transduction and cancer. Curr. Biol. *19*, R234–R241.

Reim, G., Mizoguchi, T., Stainier, D.Y., Kikuchi, Y., and Brand, M. (2004). The POU domain protein spg (pou2/Oct4) is essential for endoderm formation in cooperation with the HMG domain protein casanova. Dev. Cell *6*, 91–101.

Schoeftner, S., Sengupta, A.K., Kubicek, S., Mechtler, K., Spahn, L., Koseki, H., Jenuwein, T., and Wutz, A. (2006). Recruitment of PRC1 function at the initiation of X inactivation independent of PRC2 and silencing. EMBO J. *25*, 3110–3122.

Sharov, A.A., Dudekula, D.B., and Ko, M.S. (2005). A web-based tool for principal component and significance analysis of microarray data. Bioinformatics *21*, 2548–2549.

Sharov, A.A., Masui, S., Sharova, L.V., Piao, Y., Aiba, K., Matoba, R., Xin, L., Niwa, H., and Ko, M.S. (2008). Identification of Pou5f1, Sox2, and Nanog downstream target genes with statistical confidence by applying a novel algorithm to time course microarray and genome-wide chromatin immunoprecipitation data. BMC Genomics *9*, 269.

Smith, A.G., Heath, J.K., Donaldson, D.D., Wong, G.G., Moreau, J., Stahl, M., and Rogers, D. (1988). Inhibition of pluripotential embryonic stem cell differentiation by purified polypeptides. Nature *336*, 688–690.

Struhl, K. (1999). Fundamentally different logic of gene regulation in eukaryotes and prokaryotes. Cell *98*, 1–4.

Takahashi, K., and Yamanaka, S. (2006). Induction of pluripotent stem cells from mouse embryonic and adult fibroblast cultures by defined factors. Cell *126*, 663–676.

Wang, Y., Chen, J., Hu, J.L., Wei, X.X., Qin, D., Gao, J., Zhang, L., Jiang, J., Li, J.S., Liu, J., et al. (2011a). Reprogramming of mouse and human somatic cells by high-performance engineered factors. EMBO Rep. *12*, 373–378.

Wang, X., Bai, L., Bryant, G.O., and Ptashne, M. (2011b). Nucleosomes and the accessibility problem. Trends Genet. *27*, 487–492.

Williams, R.L., Hilton, D.J., Pease, S., Willson, T.A., Stewart, C.L., Gearing, D.P., Wagner, E.F., Metcalf, D., Nicola, N.A., and Gough, N.M. (1988). Myeloid leukaemia inhibitory factor maintains the developmental potential of embryonic stem cells. Nature *336*, 684–687.

Yeap, L.S., Hayashi, K., and Surani, M.A. (2009). ERG-associated protein with SET domain (ESET)-Oct4 interaction regulates pluripotency and represses the trophectoderm lineage. Epigenetics Chromatin *2*, 12.

Ying, Q.L., Nichols, J., Chambers, I., and Smith, A. (2003). BMP induction of Id proteins suppresses differentiation and sustains embryonic stem cell self-renewal in collaboration with STAT3. Cell *115*, 281–292.

Ying, Q.L., Wray, J., Nichols, J., Batlle-Morera, L., Doble, B., Woodgett, J., Cohen, P., and Smith, A. (2008). The ground state of embryonic stem cell self-renewal. Nature *453*, 519–523.

Yuan, P., Han, J., Guo, G., Orlov, Y.L., Huss, M., Loh, Y.H., Yaw, L.P., Robson, P., Lim, B., and Ng, H.H. (2009). Eset partners with Oct4 to restrict extraembryonic trophoblast lineage potential in embryonic stem cells. Genes Dev. *23*, 2507–2520.

Zamparini, A.L., Watts, T., Gardner, C.E., Tomlinson, S.R., Johnston, G.I., and Brickman, J.M. (2006). Hex acts with beta-catenin to regulate anteroposterior patterning via a Groucho-related co-repressor and Nodal. Development *133*, 3709–3722.

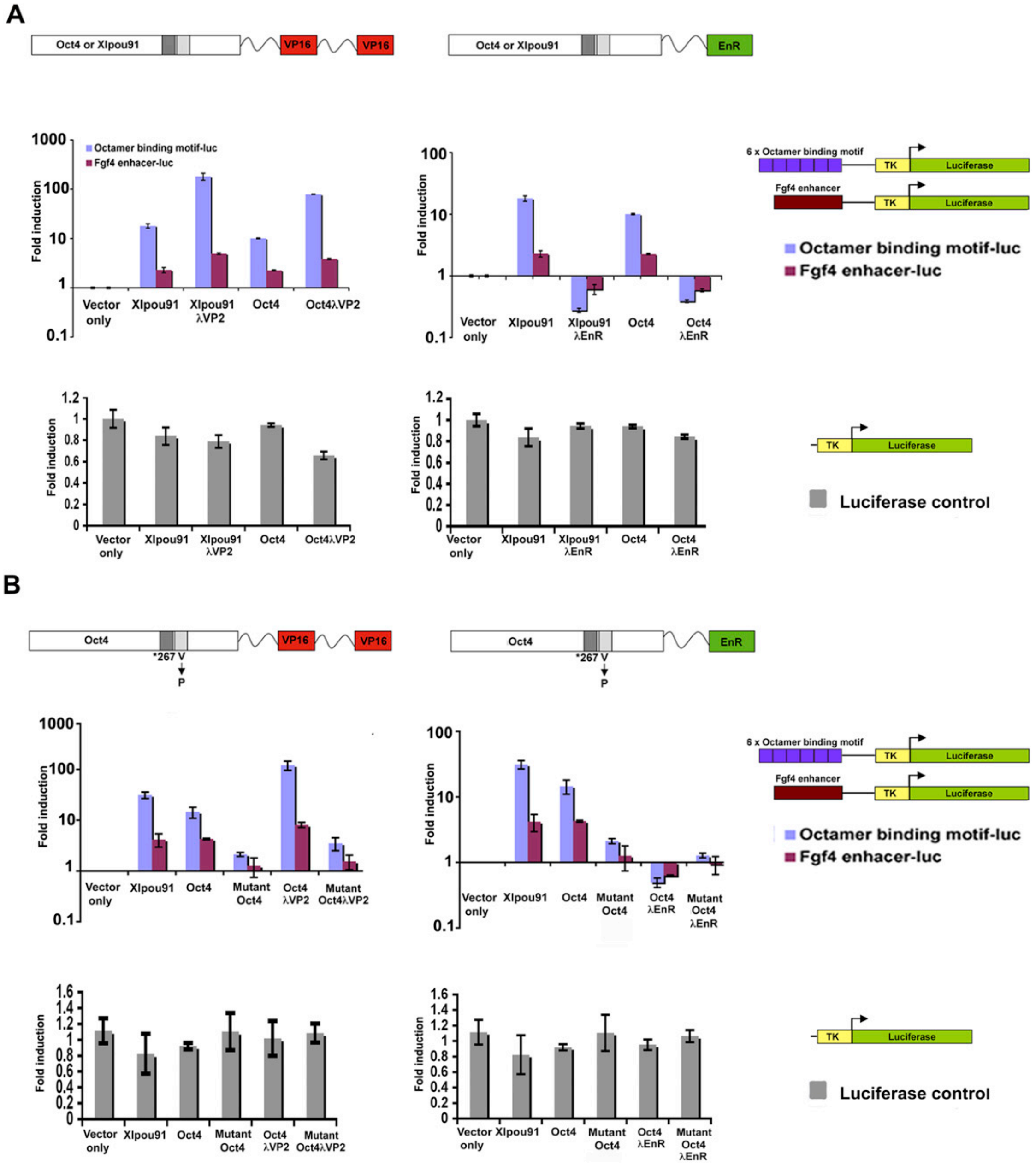

Figure S1. Transcriptional Activity of Oct4 and Xlpou91 Fusion Proteins, Related to Table 1

(A) Activity of Oct4 and Xlpou91, λVP2 and λEnR fusion proteins on different reporter genes. Different octamer recognition sites are positioned upstream of the minimal *thymidine kinase (TK)* promoter driving luciferase. The reiterated octamer reporter contains 6 copies of the octamer-binding motif derived from the mouse immunoglobulin heavy gene enhancer and the Fgf4 reporter contains the 460 bp enhancer region that includes both Sox2 and Oct4 binding sites. Co-transfection of activator fusions alongside these reporters in ZHBTc4 ESCs lead to induction of luciferase, whereas the repressor fusions suppress the basal level of the TK promoter. None of the tested fusions had any activity on a reporter lacking octamer recognition elements.

(B) DNA-binding deficient Oct4V267P fusions to either λVP2 or λEnR had no activity on any of the reporters tested in A. All transfections were done 24 hr following the plating of ZHBTc4 ESCs in the presence of Tc. This is to insure that all Oct4 activity originates from the exogenous expression vectors. Fold induction represents the increase in reporter transcription compared with the control. Data represent the mean value of two independent experiments.

Cell PRESS
Open ACCESS

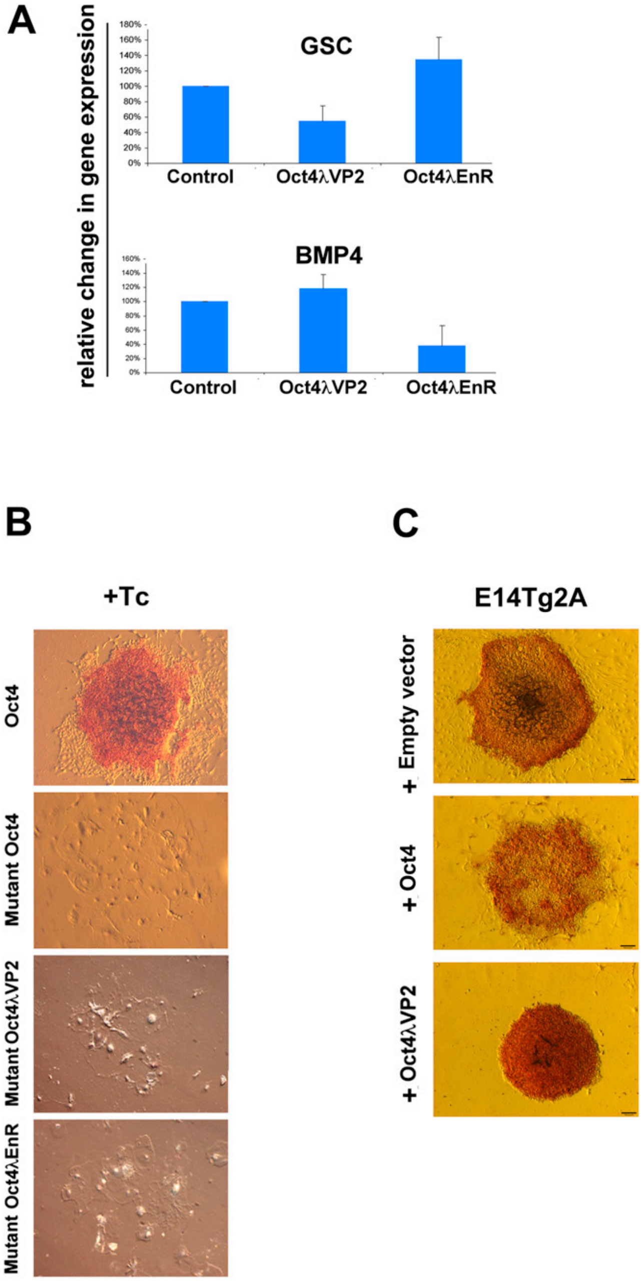

Figure S2. PouV Activator Proteins, but Not DNA Binding Mutants, Support the Undifferentiated State, Related to Figure 1

(A) qRT-PCR quantification of *Gsc* and *BMP4* RNA levels in response to PouV fusion protein overexpression in *Xenopus* embryos. Total RNA was collected from stage 10.25 embryos following injection of mRNA encoding PouV fusion proteins at the two cell stage. Gene expression levels were normalized to those from the control embryos.

(B) Oct4V267P, Oct4V267PλVP2 and Oct4V267PλEnR do not support undifferentiated colony formation in the absence of Oct4. ZHBTc4 ESCs were transfected with vectors expressing the indicated PouV fusion under the control of the CAG promoter in the presence of Tc. Plates were stained with alkaline phosphatase (AP) ten days following transfection and representative colonies are shown.

(C) Overexpression of Oct4λVP2 in wild-type E14Tg2A ESCs. Undifferentiated colony morphologies were observed when Oct4λVP2 was introduced into the E14Tg2A ESC line, despite the presence of endogeneous Oct4. These colonies are morphologically similar to those obtained from transfection of the ZHBTc4 ESCs with PouV activator proteins in the absence of Tc. E14Tg2A ESCs were transfected with vectors expressing the indicated proteins under the control of the CAG promoter. Plates were stained with AP ten days following transfection and representative colonies are shown.

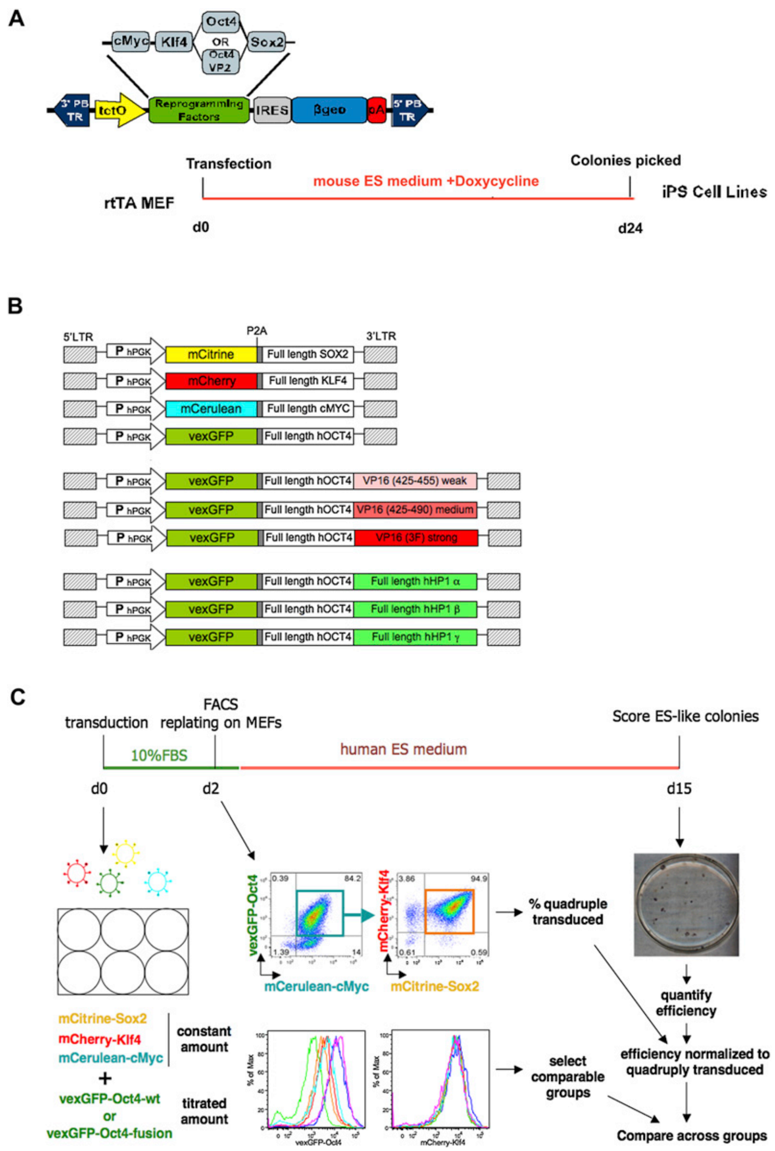

Figure S3. The Approach to iPSC Generation in Both Mouse and Human Cells, Related to Figure 2

(A) Schematic diagram of the strategy used to generate mouse iPSCs from mouse embryonic fibroblasts (MEFs) using Oct4λVP2 in place of Oct4. MEFs were derived from mice carrying rtTA in the Rosa26 locus and were transfected with *piggy*Bac transposon containing *c-Myc, Klf4, Sox2* and either wild-type *Oct4* or *Oct4λVP2* expressed from a single transcript. Transfected cells were cultured in the presence of doxycycline (Dox) to induce the expression of the four factors. Colonies were picked 24 days after transfection and expanded.

(B) Diagram depicting the construction of the human OCT4 activator and repressor forms. Separate lentiviral constructs encoding full length human OCT4, SOX2, KLF4 and c-MYC that are linked by a P2A peptide to vexGFP, mCitrine, mCherry and mCerulean, respectively, were used. Activator forms of OCT4 were generated by fusing its C terminus to different versions of the VP16 activation domain: a weak VP16 fusion (425-455aa), a medium strength VP16 (425-490aa) and a strong VP16 ('3F'; 3 tandem repeats of PADALDDFDLDML). Repressor fusions of OCT4 were made by fusing full length HP1α, HP1β or HP1γ to the C terminus of OCT4.

(C) Schematic diagram of the strategy used to generate iPSCs using OCT4 activator and repressor forms from human embryonic fibroblast (MRC-5) (from ATCC). MRC-5 were transduced with supernatants of the four lentiviral vectors. In order to achieve accurate comparisons for reprogramming efficiency, flow cytometry was used three days following transfection to identify cells with similar expression levels of the four factors.

The experiment shown in Figure 2C was performed as follows: MRC-5 were transduced with wild-type OCT4 or OCT4-VP16 at 3 different multiplicities of infection (MOIs) for each vector. Flow cytometry was used to measure expression of OCT4 (by means of measuring the mean fluorescence intensity (MFI) of vexGFP co-expressed stoichiometrically with OCT4 or OCT4-VP16 through a P2A peptide). In order to control for levels of expression - so that the differences in re-programming efficiency can only be attributed to the presence versus absence of the VP16 domain - MFI was converted to arbitrary units and depicted as "relative OCT4 expression" (*x* axis). Higher OCT4-VP16 expression (corresponding to 3x arbitrary units) was not experimentally feasible, as it resulted in excessive cell death immediately after (within 48h) transduction (vector-mediated toxicity, possibly attributable to VSVG capsid protein toxicity used for lentiviral vector packaging).

Cell PRESS Open ACCESS

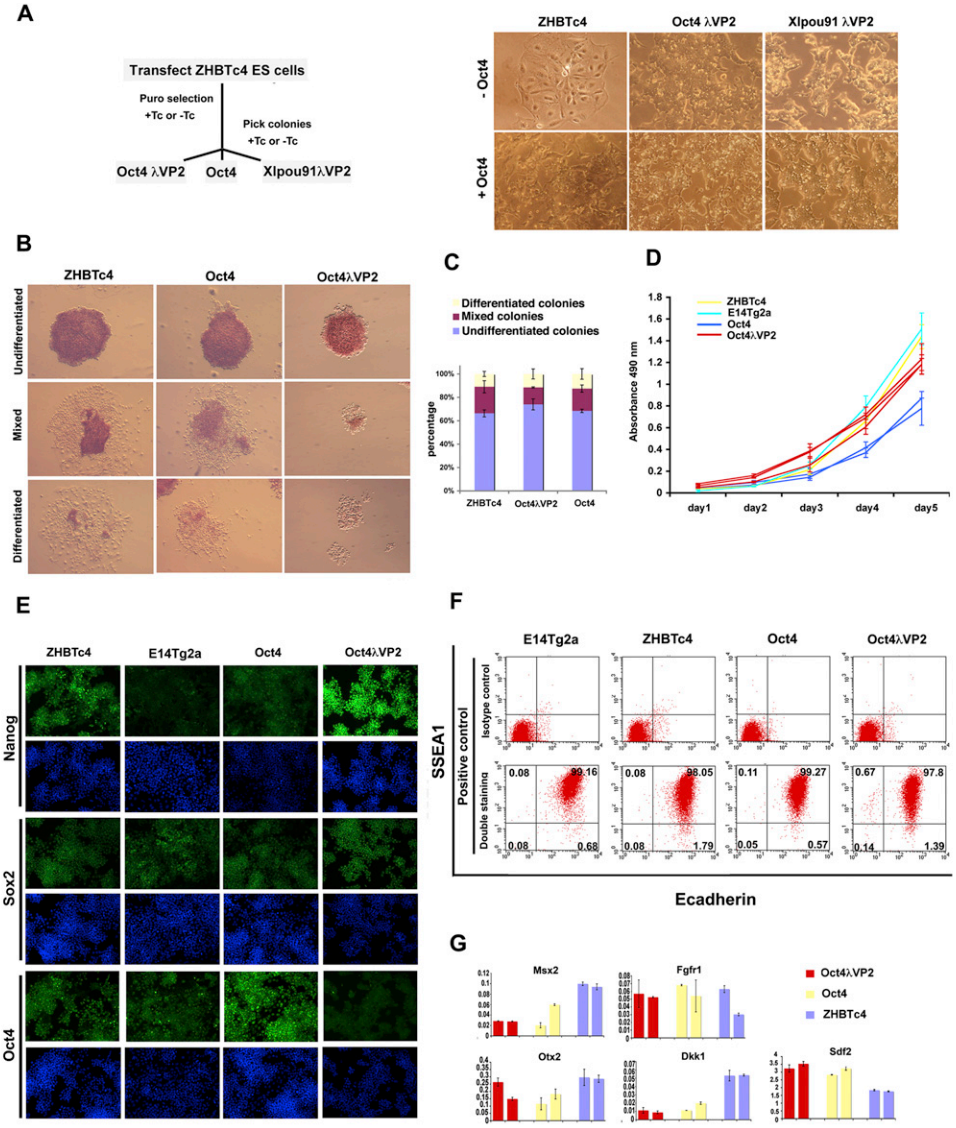

Figure S4. Characterization of PouV Activator-Rescued ESC Lines, Related to Figure 3

(A) The derivation of clonal ESC lines supported by Oct4λVP2 and Xlpou91λVP2 in place of Oct4. Following the electroporation of *Oct4λVP2* and *Xlpou91λVP2* constructs in ZHBTc4 cells, puromycin selection was applied for nine days in the presence of Tc (-*Oct4*). The rescued colonies were then picked and expanded under the same conditions. The figure (right hand panel) shows the morphology of Oct4λVP2 and Xlpou91λVP2 expressing cell lines at passage 5 (p5) in the presence of continued Tc, compared to the morphology obtained as a result of expansion in the presence of Oct4 expressed from the Tc regulatable transgene (+Oct4)

(B) Oct4λVP2 supports ESC clonal self-renewal at similar levels to wild-type Oct4 in the presence of LIF and serum. The indicated cell lines were plated at clonal density (60 cells/cm2), and cultured for 6 days under standard self-renewal conditions (LIF and serum) then stained for AP (red). The figure shows examples of the colony morphology present in each cell line.

(C) Oct4λVP2-supported cell lines give rise to completely undifferentiated colonies at a similar frequency as control ESC lines. Colonies were classified into three categories: Uniformly AP positive undifferentiated colonies, mixed colonies containing AP positive and negative cells, and AP negative differentiated colonies. Data represents average values from two independent clones for each cell line.

(D) Oct4λVP2 rescued cell lines proliferate at a similar rate to wild-type and control (Oct4 rescued ZHBTc4) ESCs. Cell growth was measured by an MTS assay and is plotted on the *y*-axis against time (days) on the *x*-axis. Data represent one of two experiments in which each data point was performed in triplicate.

(E) Oct4λVP2 supported ESC lines have increased Nanog and normal Sox2 expression, despite reduced levels of Oct4λVP2. The figure depicts typical images based on data from three independent clones of activator supported cell lines cultured under standard ESC self-renewal conditions (Serum and LIF) and immunostained for Oct4, Nanog and Sox2 proteins. DAPI staining is shown for each cell line and antibody.

(F) Oct4λVP2 supported cell lines express normal levels of ESC associated cell surface markers SSEA-1 and E-cadherin. The figure shows representative data from one of three independent clones of Oct4λVP2 and Oct4 cell lines that were grown under self-renewal conditions (serum and LIF) and subjected to analysis by flow cytometry for SSEA-1 and E-cadherin. ZHBTc4 and E14Tg2A cell lines were used as positive controls.

(G) Expression of differentiation genes in Oct4λVP2 rescued cells. Expression of differentiation-related genes was measured by qRT-PCR. Expression levels of all analyzed genes were normalized to *TBP* levels.

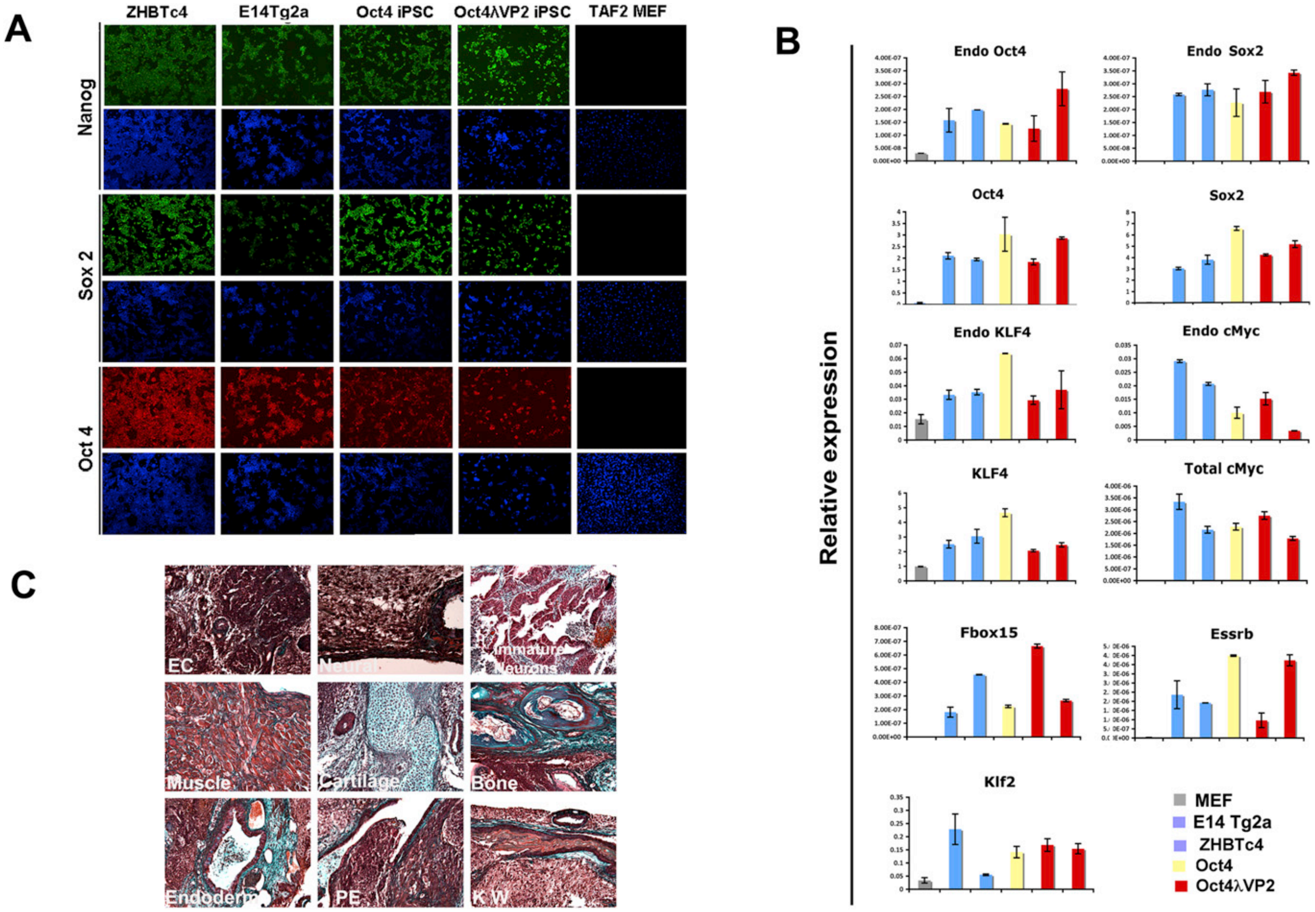

Figure S5. Characterization of Mouse iPSC Lines Generated Using Either Oct4 or Oct4λVP2, Related to Figure 3

(A) Expression of Oct4, Nanog and Sox2 in iPSCs generated by reprogramming with either Oct4 or Oct4λVP2. Expanded wild type Oct4 and Oct4λVP2 iPSC-lines were cultured in the absence of Dox (absence of the original exogenous reprogramming factors) and factor independent expression of endogenous Oct4 and Sox2 as well as Nanog was confirmed by immunofluorescence. DAPI nuclear staining (blue field) images are shown for each antibody staining.

(B) Reactivation of the endogenous transcripts for pluripotency genes in Oct4λVP2 iPSCs. Cells were cultured under self-renewal conditions and RNA was collected for qRT-PCR. The expression levels of the endogenous *Klf4*, *Oct4*, *Sox2* and *c-Myc* and a number of other pluripotency markers are shown.

(C) iPSCs generated by Oct4λVP2 are pluripotent. Cells were subcutaneously transplanted into nude mice. After 4 weeks, tumors were sectioned and stained with hematoxylin and eosin staining. Tumours contained EC cells, differentiated neural tissue, immature neurons, muscle tissue, cartilage, bone, endoderm tissue, pulmonary epithelium (PE) and keratinised epithelium (KW).

Cell PRESS
Open ACCESS

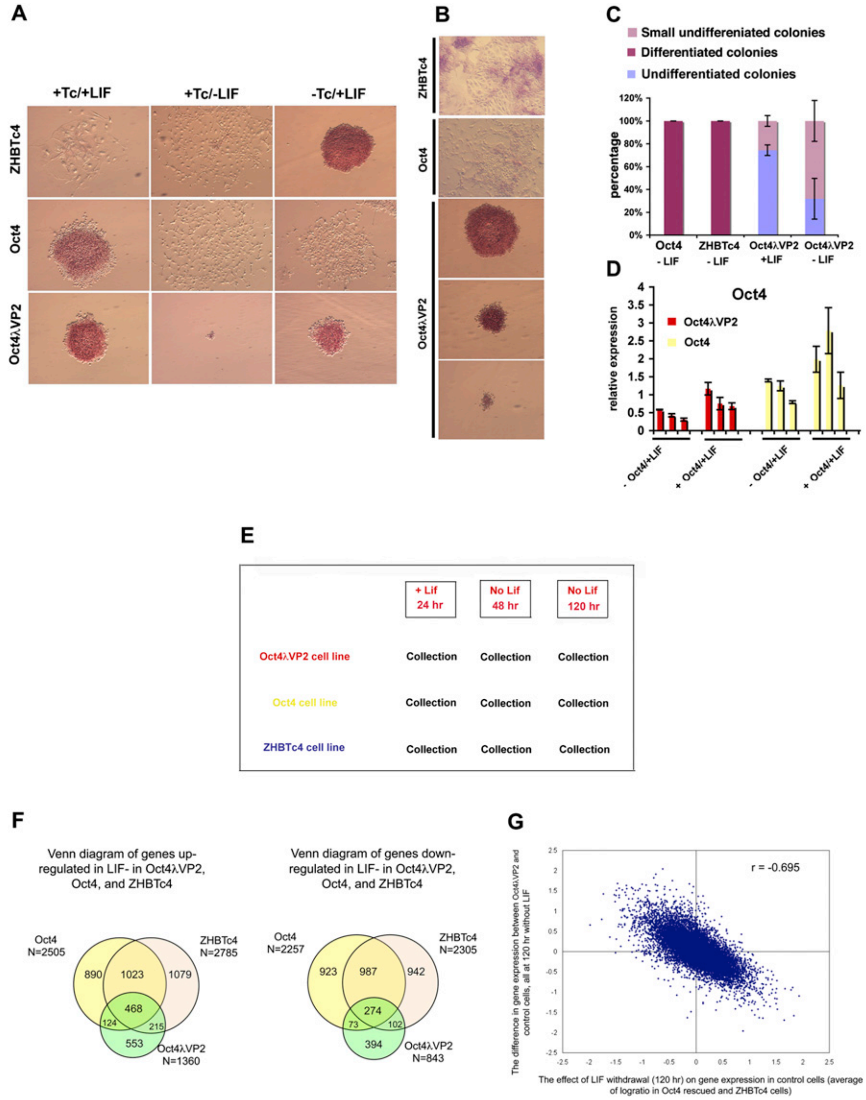

Figure S6. Oct4λVP2 Supports Self-Renewal under Differentiation Inducing Conditions, Related to Figure 4

(A) Oct4λVP2 supports clonal self-renewal in the absence of LIF or presence of additional Oct4. The indicated cell lines were plated at clonal density in the presence or absence of LIF or the presence of Oct4 overexpression for 5 days and colonies stained with AP. While the Oct4λVP2 colonies are small, they remain AP positive.

(B) Slow growing Oct4λVP2-supported cells in the absence of LIF form phenotypically normal ES colonies when allowed to grow for longer periods. ESC colonies derived from Oct4λVP2-supported cell lines were allowed to grow for 15 days in the absence of LIF and were stained with AP.

(C) Quantitative analysis of LIF independent undifferentiated growth observed in Oct4λVP2-supported ESCs. Colonies were classified into three categories after 15 days of LIF independent growth: Uniformly AP positive undifferentiated colonies, mixed colonies containing AP positive and negative cells, and AP negative differentiated colonies. In the case of Oct4λVP2 cell lines, colonies were classified into AP positive undifferentiated normal size and small colonies. Data represents average values from two independent clones from each cell line.

(D) Total Oct4 and Oct4λVP2 expression levels observed after reactivation of Tc regulatable Oct4 transgene. RNA was collected at day 5 of the differentiation experiments for qRT-PCR analysis. Levels of Oct4 expression are relative to *TBP*.

(E) Schematic representation of the experimental design and micro-array time points used to assess global gene expression during LIF withdrawal. Oct4λVP2 cells and control cell lines were plated at low density under self-renewing conditions for 24 hr and RNA collected at this time point. The medium was then changed to medium without LIF and cells were allowed to differentiate. RNA was collected after 48h and 120h. Two clones from each cell line were used as biological replicates.

(F) Venn diagrams showing that only a minority of differentiation specific genes are upregulated in Oct4λVP2 cells during LIF withdrawal and only a minority of the ESC gene-expression set normally downregulated during LIF withdrawal is affected in Oct4λVP2 cells.

(G) Genes normally regulated by LIF withdrawal correlate very well with genes upregulated in Oct4λVP2-supported ESCs in the absence of LIF. The plot compares the effect of LIF withdrawal on gene expression after 120 hr of differentiation to the gene expression differences between Oct4λVP2 cells and control cells at this same time point. There is a strong negative correlation ($r = -0.695$, $t = 153$, $p < 0.0001$) between the average log ratio of LIF withdrawal effects in control cells and the difference in gene expression between Oct4λVP2-rescued cells and control cells.

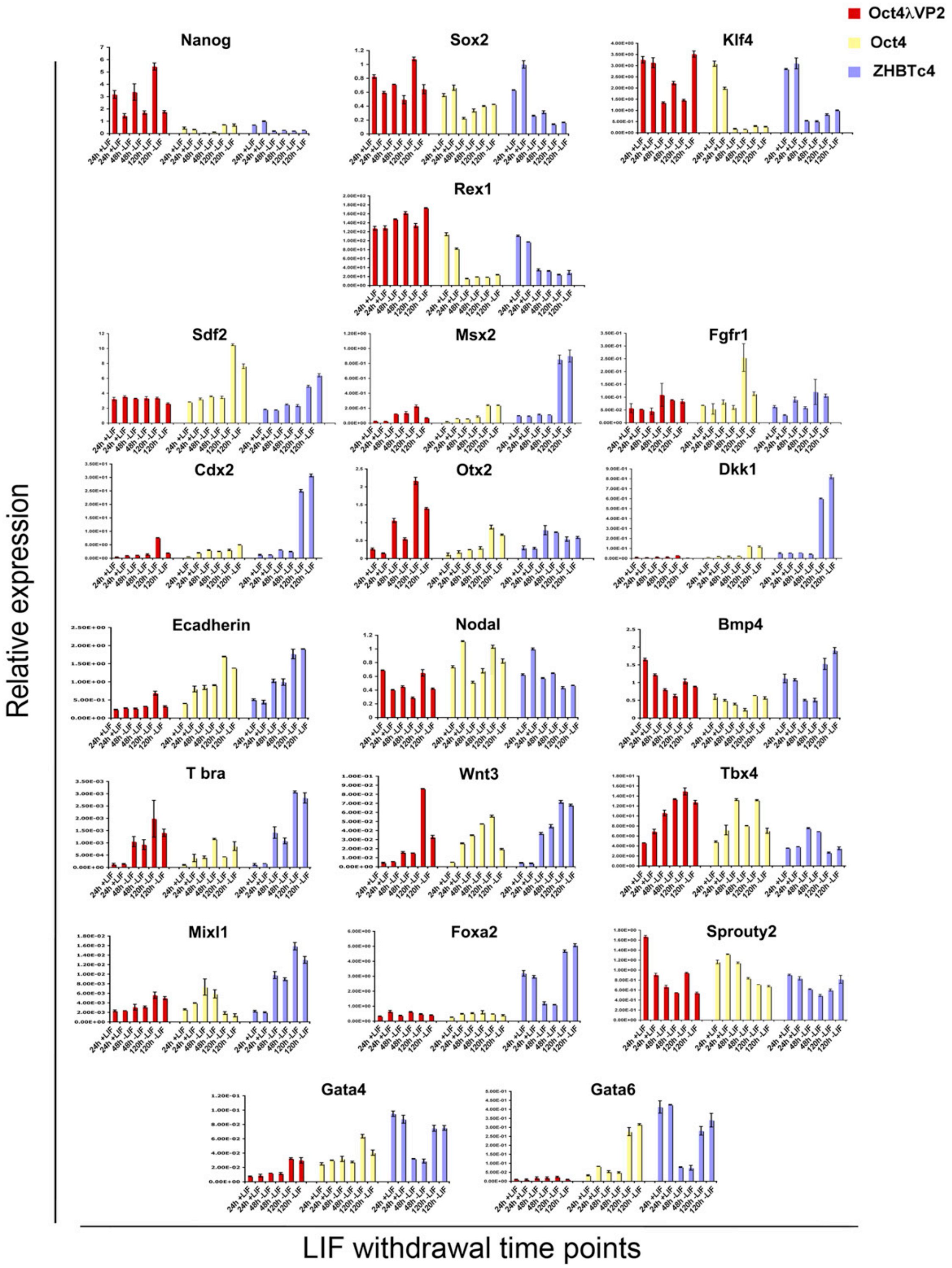

Figure S7. Oct4λVP2 Supported ESCs Retain Significant Pluripotent Character in LIF Withdrawal, Related to Figure 4

Gene expression in Oct4λVP2 supported cells during LIF withdrawal was assessed by qRT-PCR. Total RNA was collected during the LIF withdrawal time course. qRT-PCR was used to assess the expression of key pluripotency, mesoderm and endoderm genes in Oct4λVP2 cells during LIF withdrawal. Duplicates bars in each condition represent independent clones. Expression levels were normalized to TBP levels.

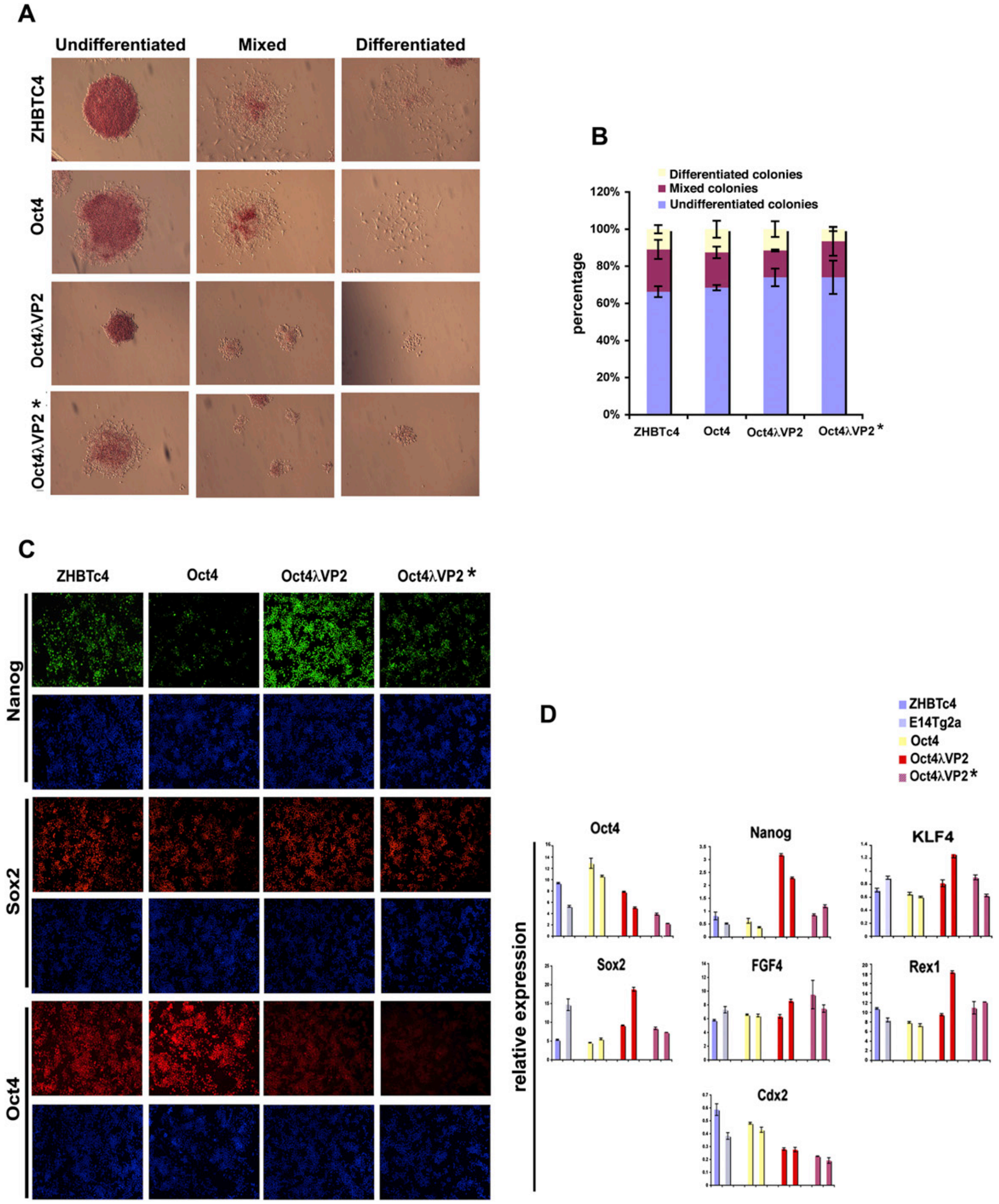

Figure S8. Cell Lines Supported by Reduced Levels of Oct4λVP2 Have a Normal ESC Phenotype, Related to Figure 4

(A) Examples of colony morphology present in each cell line. Oct4λVP2* cell lines derived in lower levels of puromycin contain differentiated cells at the periphery. The indicated cell lines were plated at clonal density (60 cells/cm^2), and cultured for 6 days in self-renewal conditions then stained for AP activity (red).

(B) Quantification of self-renewal and differentiation rates in Oct4λVP2 versus Oct4λVP2* cell lines. Colonies were classified into three categories: Uniformly AP positive undifferentiated colonies, mixed colonies containing AP positive and negative cells, and AP negative differentiated colonies. The percentages of the different colony categories present in each cell line are shown. Data represents average values from two independent clones from each cell line.

(C) Oct4λVP2* cell lines derived in lower levels of puromycin express lower levels of the Oct4λVP2 transgene and some pluripotency markers when compared to the original Oct4λVP2 cell line. Two clones from each cell line (except ZHBTc4) cultured under self-renewal conditions were fixed and immunostained for Oct4, Nanog and Sox2 proteins. Representative images are shown. DAPI nuclear staining (blue field) is shown alongside each antibody staining.

(D) Expression of key pluripotency network members in Oct4λVP2* cells. RNA from two independent clones was subjected to qRT-PCR analysis of expression levels of pluripotency markers and trophoblast differentiation marker Cdx2. Expression levels of all analyzed genes were normalized to *TBP* levels.
